# Late‐breaking trials in IBD

**DOI:** 10.1002/ueg2.70130

**Published:** 2025-11-03

**17:00‐18:00/Room Helsinki**

## LB01 | Efficacy and Safety of Obefazimod in Patients With Moderately to Severely Active Ulcerative Colitis: Results From Two, Phase 3, Randomized, Double‐Blind, Placebo‐Controlled, 8‐Week Induction Trials (Abtect‐1 & 2)

B. E. Sands ^1^, S. Danese^2^, L. Peyrin‐Biroulet^3^, M. C. Dubinsky^4^, T. Hisamatsu^5^, H. Tilg^6^, R. Atreya^7^, A. Armuzzi^8^, X. Treton^9^, F. Baert^10^, U. Seidler^11^, F. Cataldi^12^, D. Jacobstein^12^, C. Rabbat^12^, K. Shan^12^, G. DuVall^13^, B. Siegmund^14^, P. S. Dulai^15^, D. T. Rubin^16^, S. Vermeire^17^

^1^Division of Gastroenterology, Icahn School of Medicine at Mount Sinai, New York, New York, USA, ^2^Gastrointestinal Immunopathology, IRCCS San Raffaele Scientific Institute, Vita‐Salute San Raffaele University, Milan, Italy, ^3^Gastroenterology, INFINY Institute, INSERM NGERE, Nancy University Hospital, Vandoeuvre‐les‐Nancy, France, ^4^Mount Sinai Kravis Children's Hospital, Pediatric GI and Nutrition, New York, New York, USA, ^5^Kyorin University School of Medicine, Tokyo, Japan, ^6^Department of Medicine, Innsbruck Medical University, Innsbruck, Austria, ^7^Medical Department 1, University Erlangen‐Nuremberg, Erlangen, Germany, ^8^Gastroenterology, IBD Center, IRCCS Humanitas Research Hospital, Milan, Italy, ^9^Groupe Hospitalier Prive Ambroise Pare – Hartmann, Institut des MICI, Neuilly sur Seine, France, ^10^Department of Gastroenterology, Az Delta, Roeselare, Belgium, ^11^Medizinische Hochschule Klinik f. Gastroenterlogie, Hannover, Germany, ^12^Abivax, Paris, France, ^13^Tyler Research Institute, Tyler, USA, ^14^Med. Klinik m.S. Gastroenterologie, Infektiologie und Rheumatologie, Charité ‐ Universitätsmedizin Berlin, Berlin, Germany, ^15^Gastroenterology & Hepatology, Feinberg School of Medicine, Northwestern University, Chicago, Illinois, USA, ^16^University of Chicago Medicine, Chicago, Illinois, USA, ^17^Department of Gastroenterology, University Hospital Leuven, Leuven, Belgium

**Contact E‐Mail Address:** bruce.sands@mssm.edu

**Introduction:** Obefazimod (Obe) is an oral, once‐daily (QD), small molecule which enhances expression of microRNA‐124 and has been studied in two Phase 2 induction trials and subsequent open‐label maintenance studies [1–3] in patients (pts) with moderately to severely active ulcerative colitis (UC). Here we report efficacy and safety of two Phase 3, 8‐week, induction trials in adult pts with UC from ABTECT‐1 [NCT05507203] and ABTECT‐2 [NCT05507216].

**Aims & Methods:** The multicenter, randomized, double‐blind, placebo‐controlled ABTECT trials enrolled pts with moderate‐to‐severe UC (defined as modified Mayo score (MMS) ≥ 5, with rectal bleeding sub‐score (RBS) ≥ 1 and centrally read endoscopic score ≥ 2) who had inadequate response, loss of response, or intolerance to at least one prior therapy (with no upper limit), including corticosteroids, immunosuppressants, biologics, S1P receptor modulators and/or JAK inhibitors. Pts were randomized 2:1:1 to Obe 50 mg QD (Obe‐50), Obe 25 mg QD (Obe‐25) or placebo (PBO) for 8 weeks. The primary endpoint was clinical remission (per MMS) and secondary endpoints included clinical response, endoscopic improvement, symptomatic remission, and histo‐endoscopic mucosal improvement (HEMI).

**Results:** 1272 pts were randomized and treated in ABTECT‐1 (636) and ABTECT‐2 (636). In both trials, baseline demographics and disease characteristics were similar between groups; 45.3% and 49.3% of pts had inadequate response to one or more advanced therapies. A significantly higher proportion of pts receiving Obe‐50 (ABTECT‐1:21.7%, ABTECT‐2: 19.8%) versus PBO (2.5% and 6.3%) achieved clinical remission (Obe‐50‐PBO difference: ABTECT‐1: 19.3%, *p* < 0.0001; ABTECT‐2: 13.4%, *p* = 0.0001) and met all key secondary endpoints in both trials. A significantly higher proportion of pts receiving Obe‐25 versus PBO achieved clinical remission (Obe‐25‐PBO difference: 21.4%, *p* < 0.0001) and met all key secondary endpoints in ABTECT‐1. In a pooled analysis, both Obe‐50 and Obe‐25 met all primary and secondary endpoints with nominal significance (*p* < 0.0001) (Table 1). The overall rate of serious adverse events and treatment emergent adverse events (TEAEs) leading to study drug discontinuation for pts treated with Obe were similar to PBO. Proportions of pts who reported at least one TEAE in ABTECT‐1 were 59.4%, 46.9%, and 53.2% for Obe‐50, Obe‐25, and PBO, respectively. For ABTECT2, TEAEs occurred in 61.0%, 50.9%, and 48.4% for Obe‐50, Obe‐25, and PBO, respectively. The most frequent TEAE was headache (Obe‐50: 20.8%–25.8%; Obe‐25: 14.5%–15.6%; PBO: 5.7). The headaches were mild, transient, short in duration and not a barrier to treat as evidenced by a low discontinuation rate of 0%–1.6%. No signal was observed for serious, severe, or opportunistic infections or malignancies.

**TABLE 1**8‐week induction efficacy of obefazimod in ABTECT‐1 and ABTECT‐2 Phase 3 studies, and pooled ABTECT‐1 and ABTECT‐2^‡^

**Table:** 

Efficacy endpoints at week 8, % (*n*)	ABTECT‐1					ABTECT‐2					Pooled ABTECT‐1 and ABTECT‐2				
	PBO	Obe‐25	Obe‐50	Between group diff.	Between group diff.	PBO	Obe‐25	Obe‐50	Between group diff.	Between group diff.	PBO	Obe‐25	Obe‐50	Between group diff.	Between group diff.
	*N* = 158	*N* = 160	*N* = 318	Obe‐25 versus PBO	Obe‐50 versus PBO	*N* = 159	*N* = 159	*N* = 318	Obe‐25 versus PBO	Obe‐50 versus PBO	*N* = 317	*N* = 319	*N* = 636	Obe‐25 versus PBO	Obe‐50 versus PBO
Clinical remission	2.5 (4)	23.8 (38)	21.7 (69)	21.4 *p* < 0.0001	19.3 *p* < 0.0001	6.3 (10)	11.3 (18)	19.8 (63)	5.1 *p* = 0.1034^¥^	13.4 *p* = 0.0001	4.4 (14)	17.6 (56)	20.8 (132)	13.2^a^ *p* < 0.0001	16.4^a^ *p* < 0.0001
Clinical response	28.5 (45)	65.6 (105)	61.0 (194)	37.2 *p* < 0.0001	32.6 *p* < 0.0001	33.3 (53)	53.5 (85)	63.2 (201)	20.1 *p* = 0.0002^¥^	29.6 *p* < 0.0001	30.9 (98)	59.6 (190)	62.1 (395)	28.6^a^ *p* < 0.0001	31.2^a^ *p* < 0.0001
Endoscopic improvement†	5.7 (9)	37.5 (60)	33.3 (106)	32.0 *p* < 0.0001	27.8 *p* < 0.0001	10.1 (16)	22.0 (35)	35.5 (113)	12.0 *p* = 0.0029^¥^	25.4 *p* < 0.0001	7.9 (25)	29.8 (95)	34.4 (219)	21.9^a^ *p* < 0.0001	26.6^a^ *p* < 0.0001
HEMI	3.2 (5)	23.8 (38)	23.0 (73)	20.7 *p* < 0.0001	20.0 *p* < 0.0001	7.5 (12)	13.2 (21)	23.9 (76)	5.7 *p* = 0.0932^¥^	16.4 *p* < 0.0001	5.4 (17)	18.5 (59)	23.4 (149)	13.2^a^ *p* < 0.0001	18.2^a^ *p* < 0.0001
Symptomatic remission*^,^†	17.7 (28)	42.5 (68)	41.2 (131)	24.9 *p* < 0.0001	23.7 *p* < 0.0001	22.0 (35)	33.3 (53)	40.3 (128)	11.4 *p* = 0.0227	18.0 *p* < 0.0001	19.9 (63)	37.9 (121)	40.7 (259)	18.1^a^ *p* < 0.0001	21.0^a^ *p* < 0.0001

NRI is used for subjects with missing outcome at week 8 and subjects reporting any IE prior to week 8. % Difference is for Obe minus placebo and is based on estimated common risk difference using the Mantel‐Haenszel weights adjusting for the randomization stratification factors: inadequate response to advanced therapies (yes/no), baseline oral corticosteroids usage (yes/no), and region (Japan/rest of world) [ABTECT‐2 only]. *p*‐values are two sided. ^a^ for pooled analysis, all *p*‐values are nominal. Clinical remission: stool frequency sub‐score (SFS) ≤ 1, rectal bleeding sub‐score (RBS) = 0 and endoscopic sub‐score ≤ 1. Clinical response: decrease from baseline in the MMS ≥ 2 points and ≥ 30% from baseline, plus a decrease in RBS ≥ 1 or an absolute RBS ≤ 1. Endoscopic improvement: endoscopic sub‐score ≤ 1. Symptomatic remission: RBS = 0, SFS < 1. HEMI: combination of histologic improvement (Geboes histologic score ≤ 3.1) and endoscopic improvement (endoscopy sub‐score ≤ 1). * Symptomatic remission was an “other secondary” endpoint, not multiplicity controlled, for the FDA protocol. † Endoscopic improvement/symptomatic remission were co‐primary endpoints for the EMA protocol and were met by both doses in both trials. ‡ Hierarchical testing strategy was used starting with 50 mg for the primary endpoint followed by the key secondary endpoints; the 25 mg was subsequently tested for the primary endpoint followed by the key secondary endpoints. ¥ 25 mg did not meet the primary endpoint at week 8 in ABTECT‐2 in the FDA testing protocol, therefore *p*‐values for key secondary endpoints for the 25 mg arm in ABTECT‐2 are nominal.

**Conclusion:** In both ABTECT induction trials, primary and secondary endpoints were met; obe treatment led to statistically significant improvements in clinical, endoscopic, symptomatic and combined endoscopic‐histologic endpoints at week 8. Obe was well tolerated with no new safety signals identified.

**References**

1. S. Vermeire, V. Solitano, L. Peyrin‐Biroulet, et al., “Obefazimod: A First‐in‐Class Drug for the Treatment of Ulcerative Colitis,” *Journal of Crohn's and Colitis* 17, no. 10 (2023): 1689–1697, 10.1093/ecco‐jcc/jjad067.
2. S. Vermeire, B. E. Sands, H. Tilg, et al., “ABX464 (Obefazimod) for Moderate‐to‐Severe, Active Ulcerative Colitis: A Phase 2b, Double‐Blind, Randomised, Placebo‐Controlled Induction Trial and 48 Week, Open‐Label Extension,” *Lancet Gastroenterology & Hepatology* 7, no. 11 (2022): 1024–1035, 10.1016/S2468‐1253(22)00233‐3.
3. S Vermeire, J Nitcheu, P Gineste, A Flatres, J Santo, D Scherrer, et al., “Obefazimod in Patients With Moderate‐to‐Severely Active Ulcerative Colitis: Efficacy and Safety Analysis From the 96‐Week Open‐Label Maintenance Phase 2b Study,” *Journal of Crohn's & Colitis* 19, no. 5 (2025): jjaf074, 10.1093/ecco‐jcc/jjaf074.

**Disclosure: Authors reports following potential conflicts of interest**.

**BES:** Abbvie, Abivax, Adiso Therapeutics, Agomab, Alimentiv, Amgen, AnaptysBio, Arena Pharmaceuticals, Artugen Therapeutics, Astra Zeneca, Biolojic Design, Biora Therapeutics, Boehringer Ingelheim, Bristol Myers Squibb, Celltrion, Equillium, Enthera, Enveda Biosciences, Evommune, Ferring, Fzata, Galapagos, Genentech (Roche), Gilead Sciences, GlaxoSmithKline, Gossamer Bio, Imhotex, Index Pharmaceuticals, Innovation Pharmaceuticals, Kaleido, Kallyope, Lilly, Merck & Co., Microba, Microbiotica, Mitsubishi Tanabe, Mobius Care, Morphic Therapeutics, MRM Health, Nexus Therapeutics, Nimbus Discovery, Odyssey Therapeutics, OSE Immunotherapeutics, Janssen, Palisade Bio, Pfizer, Protagonist Therapeutics, Q32 Bio, Rasayana Therapeutics, Recludix Therapeutics, Reistone Biopharma, Sanofi, Sorriso Therapeutics, Spyre Therapeutics, Takeda, Target RWE,Teva, TLL Pharmaceutical, Tr1X, Trex Bio, Union Therapeutics, Ventyx Biosciences.

**SV:** AbbVie, Abivax, AbolerIS Pharma, AgomAb, Alimentiv, Arena Pharmaceuticals, AstraZeneca, Avaxia, BMS, Boehringer Ingelheim, Celgene, CVasThera, Cytoki Pharma, Dr Falk Pharma, Ferring, Galapagos, Genentech‐Roche, Gilead, GSK, Hospira, Imidomics, Janssen, J&J, Lilly, Materia Prima, MiroBio, Morphic, MrMHealth, Mundipharma, MSD, Pfizer, Prodigest, Progenity, Prometheus, Robarts Clinical Trials, Second Genome, Shire, Surrozen, Takeda, Theravance, Tillots Pharma AG, Zealand Pharma.

**LPB:** Abbvie, Abivax, Adacyte, Alimentiv, Amgen, Applied Molecular Transport, Arena, Banook, Biogen, BMS, Celltrion, Connect Biopharm, Cytoki Pharma, Enthera, Ferring, Fresenius Kabi, Galapagos, Genentech, Gilead, Gossamer Bio, GSK, IAC Image Analysis, Index Pharmaceuticals, Inotrem, Janssen, Lilly, Medac, Mopac, Morphic, MSD, Nordic Pharma, Novartis, Oncodesign Precision Medicine, ONO Pharma, OSE Immunotherapeuthics, Pandion Therapeuthics, Par' Immune, Pfizer, Prometheus, Protagonist, Roche, Samsung, Sandoz, Sanofi, Satisfay, Takeda, Telavant, Theravance, Thermo Fischer, Tigenix, Tillots, Viatris, Vectivbio, Ventyx, Ysopia.

**SD:** AbbVie, Ferring, Hospira, Johnson & Johnson, Merck, MSD, Takeda, Mundipharma, Pfizer Inc., Tigenix, UCB Pharma, Vifor, Biogen, Celgene, Allergan, Celltrion, Sandoz, Boehringer Ingelheim.

**PSD:** Abbvie, Abivax, Adiso, Alimentiv, Bristol Meyer Squibb, Celltrion, Genentech, Geneoscopy, Janssen, Pfizer, Takeda.

**MD:** Abbvie, Abivax, Arena Pharmaceuticals, Astra Zeneca, Boehringer Ingelheim International GmbH, Bristol‐Meyer Squibb, Eli Lilly and Company, F. Hoffmann‐La Roche Ltd, Genentech Inc., Gilead, Janssen Pharmaceuticals, Merck, Pfizer Inc., Prometheus Biosciences, Takeda Pharmaceuticals.

**HT:** Abbvie, Abivax, Dr Falk Pharma, ferring, Galapagos, Microbiotica, MSD, Pfizer, Takeda.

**BS:** AbbVie, Abivax, Boehringer Ingelheim, Bristol Myers Squibb, Dr. Falk Pharma, Eli Lilly, Endpoint Health, Falk, Galapagos, Gilead, Janssen, Landos, Materia Prima, PredictImmune, Pfizer, and Takeda; AlfaSigma, CED Service GmbH, MSD, Ferring, Galapagos, Tr1x bio.

**TH:** Mitsubishi Tanabe Pharma Corporation, EA pharma Co. Ltd., AbbVie GK, JIMRO Co. Ltd., Zeria Pharmaceutical Co. Ltd., Kyorin Pharmaceutical Co. Ltd., Nippon Kayaku Co. Ltd., Takeda Pharmaceutical Co. Ltd., Pfizer Inc., Mochida Pharmaceutical Co. Ltd., Boston Scientific Corporation, Kissei Pharmaceutical Co. Ltd., Janssen Pharmaceutical K.K., Pfizer Inc., Eli Lilly, Gilead Sciences, Bristol Myers Squibb, Abivax.

**XT:** Celltrion, AbbVie, Johnson & Johnson, Lilly, Takeda, Alpha Sigma, Dr. Falk, Abivax, Biogen, Fresenius Kabi, MSD, Pfizer, Tillotts, and Thabor Therapeutics.

**RA:** AbbVie, Abivax, AstraZeneca, Bristol‐Myers Squibb, Celltrion Healthcare, Galapagos, Johnson&Johnson, Lilly, MSD, Pfizer, and Takeda Pharma.

**US:** AbbVie, Abivax, Amgen, Galapagos, Janssen, Eli Lilly, Boehringer Ingelheim, Bristol Myers Squibb, Celgene, Gilead Sciences, Pfizer, Roche, Takeda Pharmaceuticals.

**AA:** AbbVie, Abivax, AG Pharma, Alfa Sigma, Astra Zeneca, Biogen, Boehringer Ingelheim, Bristol‐Myers Squibb, Celltrion, Eli‐Lilly, Enthera, Ferring, Galapagos, Gilead, Giuliani, Janssen, Lionhealth, MSD, Nestlé, Novartis, Pfizer, Protagonist Therapeutics, Roche, Samsung Bioepis, Sanofi, Sandoz, Takeda, Teva Pharmaceuticals, Tillots Pharma.

**FB:** AbbVie, Amgen, Eurogenerics, J&J, Arena Pharmaceuticals, Celltrion, Ferring, Galapagos, Janssen, Merck Sharp & Dohme, Pfizer Inc., Takeda, Celgene, Fresenius Kabi, Sandoz.

**DTR:** Abbvie, Abivax SA, Altrubio, Athos Therapeutics Inc., Bristol‐Myers Squibb, Celltrion, Connect BioPharma, Eli Lilly & Co., Genentech (Roche) Inc., Iterative Health, Janssen Pharmaceuticals, Johnson & Johnson, Merck & Co., Odyssey Therapeutics, Pfizer, Sanofi, Spyre, Takeda Pharmaceuticals, Vedanta Biosciences, and Ventyx.

## LB02 | Donor Selection and Anaerobic Processing of Faecal Microbiota Transplantion for the Treatment of Ulcerative Colitis: Results From a Multi‐Center Randomized Controlled Trial (Turn2 Study)

F. Zwezerijnen‐Jiwa ^1,2^, M. Benard^1,3^, M. van der Spek^1^, M. Davids^4,5^, T. Middelburg^1^, M. C. Visschedijk^6^, M. Lowenberg^7^, B. Oldenburg^8^, B. Rethans^1^, G. R. D'Haens^1,9^, R. K. Weersma^10^, C. Y. Ponsioen^11^

^1^Department of Gastroenterology and Hepatology, Amsterdam UMC, Amsterdam, the Netherlands, ^2^Tytgat Institute for Liver and Intestinal Research, Amsterdam Gastroenterology Endocrinology and Metabolism, Amsterdam, the Netherlands, ^3^Inflammatory Bowel Disease Centre, Amsterdam University Medical Center, Amsterdam, the Netherlands, ^4^Microbiota Center Amsterdam (MiCA), Amsterdam, the Netherlands, ^5^Department of Experimental Vascular Medicine, Amsterdam University Medical Centers, Amsterdam, the Netherlands, ^6^Gastroenterology and Hematology, University Medical Center, Groningen, the Netherlands, ^7^Gastroenterology and Hepatology, Amsterdam University Medical Center, Amsterdam, the Netherlands, ^8^Department of Gastroenterology and Hepatology, University Medical Center Utrecht, Utrecht, the Netherlands, ^9^AMC Amsterdam Inflammatory Bowel Disease Centre, Academic Medical Center, Amsterdam, the Netherlands, ^10^Department of Gastroenterology & Hepatology, University Medical Center Groningen, Groningen, the Netherlands, ^11^AMC Amsterdam Gastroenterology and Hepatology, Amsterdam, the Netherlands

**Contact E‐Mail Address:** f.jiwa@amsterdamumc.nl

**Introduction:** Faecal microbiota transplantation (FMT) has shown promise as treatment in inducing remission in patients with ulcerative colitis (UC), however effectiveness varies (1). Optimizing FMT protocols involving anaerobic stool processing to improve bacterial viability, dual‐route administration and donor selection may improve efficacy.

**Aims & Methods:** Our aim was to evaluate the effectiveness of an optimized FMT protocol using anaerobically prepared stool from pre‐selected donors in a randomized controlled multi‐center trial using dual‐route administration in patients with mild‐to‐moderately active UC. A total of 85 patients were randomized to either receive donor FMT (*n* = 44) or autologous FMT (*n* = 41) via four weekly FMTs comprising two dual‐route administrations, nasoduodenal administration combined with enema, and two single enemas. The study was performed at the Amsterdam University Medical Centre and University Medical Centre Groningen from June 2020 to January 2025. Follow‐up time is 12 months and ongoing. Donors were selected based on their microbiota profile, informed by our previous TURN trial (2, 3). The primary outcome was steroid‐free clinical remission at week 8 defined as a total adapted Mayo score of ≤ 2, with a stool frequency subscore of ≤ 1, a rectal bleeding subscore of 0, and an endoscopic subscore of ≤ 1. Open‐label extension was offered to patients in the autologous FMT group who did not reach clinical remission.

**Results:** Eighty‐five patients were randomized (mean age 43.6 years, 48% female), with 76 (89%) patients completing all four treatments. In the modified intention‐to‐treat analysis, meaning all patients that received at least one FMT treatment (*n* = 82), primary endpoint was reached in 10 of 44 (23%) patients receiving donor FMT and in 2 of 38 (5%) patients receiving autologous FMT, (two‐sided *χ* ^2^, *p* = 0.026). A further total of 26 patients received donor FMT in open‐label extension of which 21 (81%) received all four treatments with 7/26 (27%) reaching the primary endpoint. Serious adverse events occurred in 6/44 (14%) in the donor FMT group versus 4/41 (10%) the autologous group. Two events were deemed related to the FMT treatment (pneumothorax after nasoduodenal placement and overnight hospitalization for abdominal pain one‐day post FMT).

**Conclusion:** In this multi‐center randomized controlled trial, anaerobically processed, donor‐selected, dual‐route FMT demonstrated a higher likelihood of inducing clinical remission with donor FMT compared to autologous FMT at 8 weeks. The protocol proved feasible and generally safe. Larger trials are warranted to confirm efficacy and to optimize donor and patient selection strategies. ClinicalTrial.gov. Number: NCT05998213.

**References**

1. A. Imdad, N. G. Pandit, M. Zaman, et al., “Fecal Transplantation for Treatment of Inflammatory Bowel Disease,” *Cochrane Database of Systematic Reviews* 4, no. 4 (2023): CD012774, 10.1002/14651858.CD012774.pub3.
2. S. Fuentes, N. G. Rossen, M. J. van der Spek, et al., “Microbial Shifts and Signatures of Long‐Term Remission in Ulcerative Colitis After Faecal Microbiota Transplantation,” *ISME Journal* 11, no. 8 (2017): 1877–1889, 10.1038/ismej.2017.44.
3. N. G. Rossen, S. Fuentes, M. J. van der Spek, et al., “Findings From a Randomized Controlled Trial of Fecal Transplantation for Patients With Ulcerative Colitis,” *Gastroenterology* 149, no. 1 (2015):110–118.e4, 10.1053/j.gastro.2015.03.045.

**Disclosure:** The authors report no COI with regard to this study.

## LB03 | Humoral Immune Signatures in Uninflamed Colon Mucosa Associated With Anti‐Tnf Treatment Failure in Ulcerative Colitis

G. A. Walaas^1^, S. Sveaas ^1^, L. Frostvoll Kuraas^1,2^, A. Sridhar^1,2^, K. Lillebråten^1,2^, H. Fossen^1,2^, H. P. Sahlin Pettersen^1,3^, A. Flatberg^1^, A. van Beelen Granlund^1,2,3^, A. K. Sandvik^1,2^, A. E. Østvik^1,2^, T. Bruland^1,2^, I. Bakke^1,4^

^1^Department of Clinical and Molecular Medicine, NTNU ‐ Norwegian University of Science and Technology, Trondheim, Norway, ^2^Department of Gastroenterology and Hepatology, Clinic of Medicine, St. Olav's University Hospital, Trondheim, Norway, ^3^Department of Pathology, Clinic of Laboratory Medicine, St. Olav's University Hospital, Trondheim, Norway, ^4^Clinic of Laboratory Medicine, St. Olav's University Hospital, Trondheim, Norway

**Contact E‐Mail Address:** siljsvea@ntnu.no

**Introduction:** Although anti‐TNF therapy is a cornerstone treatment for moderate to severe ulcerative colitis (UC) [1], up to 40% of patients are primary non‐responders [2]. The molecular basis for non‐response in colonic mucosa remains poorly understood.

**Aims & Methods:** We aimed to identify compartment‐specific molecular signatures in colonic epithelium and lamina propria that distinguish anti‐TNF non‐responders from responders with similar disease activity before treatment. UC patients with unambiguous endoscopic response (*n* = 6) or non‐response (*n* = 6) after 6 months anti‐TNF therapy were included. Pre‐treatment inflamed and uninflamed colonic histological biopsies were laser microdissected into epithelial and lamina propria samples for total RNA sequencing. We analyzed differentially expressed genes (DEGs), gene enrichment and weighted gene co‐expression networks (WGCNA). Cellular deconvolution and pseudobulk validation were performed using a publicly available single‐cell atlas [3], and findings were further validated by immunohistochemistry.

**Results:** Inflammation elicited mucosal compartment‐specific transcriptional changes, with more alteration in the epithelium (2654 DEGs, log2 fold change (FC) > 0.3, padj < 0.05) enriched for interferon signaling and antigen presentation, compared to heightened cell‐cycle and adaptive immune responses in the lamina propria (1525 DEGs). We found no underlying differences in treatment response in either uninflamed or inflamed epithelium, nor in gene expression of TNF and its receptors in any compartment. The main difference between non‐responders and responders was in uninflamed lamina propria where we identified distinct inflammatory signatures more overlapping with inflamed tissue (395 DEGs vs. 1112 in responders) and with high expression of cell cycle genes (e.g., *TOP2A, MKI67, CDC20, CDKN1A, FZR1*). A co‐expression gene network enriched for humoral activity (log2 FC > 0.3, padj < 0.01) correlated strongly with non‐response (*r* = 0.6, padj = 0.06) and contained numerous immunoglobulin genes (*IGHA2, JCHAIN*), including antigen‐binding variable segments of heavy (*IGHV4‐34, IGHV3‐7, IGHV3‐73*) and light chains (*IGKV1‐6, IGKV1D‐17, IGLV2‐11, IGLV2‐14*), in addition to other B cell‐associated genes (*BMI1, POU2AF1, TPD52*). The cell cycle and humoral genes were systematically expressed higher in uninflamed lamina propria of non‐responders compared to responders, with the differences disappearing in inflamed lamina propria. Cellular deconvolution^3^, showed that the humoral gene network was expressed in plasma cell populations (mainly IgA), which were confirmed increased in non‐responder uninflamed lamina propria through tissue staining and gene expression. Pseudobulk analyses of uninflamed lamina propria samples from UC non‐responders (*n* = 6) and responders (*n* = 3) filtered form the independent single‐cell dataset^3^, validated the association of both cell cycle (14 of 18) and humoral (50 of 61) genes with uninflamed lamina propria in non‐responders. Also, DEGs in uninflamed lamina propria IgA plasma cells from non‐responders compared to responders in the validation dataset, were confirmed (15 of 18) in our data.

**Conclusion:** Our findings suggest that anti‐TNF non‐response in UC involves pre‐existing humoral immune activation in macroscopically uninflamed colon mucosa. This spatially resolved signature offers a potential biomarker for treatment stratification and highlights the need for personalized therapeutic approaches in UC.

**References**

1. T. Raine, S. Bonovas, J. Burisch, et al., “ECCO Guidelines on Therapeutics in Ulcerative Colitis: Medical Treatment,” *Journal of Crohn's and Colitis* 16, no. 1 (2021): 2–17, 10.1093/ecco‐jcc/jjab178.
2. S. Ben‐Horin, U. Kopylov, and Y. Chowers, “Optimizing Anti‐TNF Treatments in Inflammatory Bowel Disease,” *Autoimmunity Reviews* 13, no. 1 (2014): 24–30, 10.1016/j.autrev.2013.06.002.
3. T. Thomas, M. Friedrich, C. Rich‐Griffin, et al., “A Longitudinal Single‐Cell Atlas of Anti‐Tumor Necrosis Factor Treatment in Inflammatory Bowel Disease,” *Nature Immunology* 25, no. 11 (2024): 1–14, 10.1038/s41590‐024‐01994‐8.

**Disclosure:** Nothing to disclose.

## LB04 | Risk of Relapse After Cessation of Anti‐Tnf Therapy in Ulcerative Colitis in Corticosteroid‐Free Clinical Remission: An Individual Participant Data Meta‐Analysis (Ipd‐Ma) on 654 Patients From 8 Studies

M. Devillers ^1^, S. ten Bokkel Huinink^1^, T. Hackmann^2^, E. Steyerberg^3^, B. Oldenburg^4^, R. Mahmoud^4^, P. Molander^5^, K. Farkas^6^, G. Fiorino^7^, M. Chaparro^8^, J. Gisbert^8^, M.J. Casanova^8^, S.‐N. Hong^9^, N. Dussias^10^, A. de Vries^1^

^1^Gastroenterology and Hepatology, Erasmus MC University Medical Center, Rotterdam, the Netherlands, ^2^Department of Biomedical Data Sciences, Leiden University Medical Center, Leiden, the Netherlands, ^3^Utrecht University Medical Center, Julius Center for Health Sciences and Primary Care, Utrecht, the Netherlands, ^4^Department of Gastroenterology and Hepatology, University Medical Center Utrecht, Utrecht, the Netherlands, ^5^Abdominal Center, Gastroenterology, Helsinki University Hospital, Helsinki, Finland, ^6^1st Department of Medicine, University of Szeged, Szeged, Hungary, ^7^IBD Unit, Gastroenterology and Digestive Endoscopy, San Camillo‐Forlanini Hospital, Rome, Italy, ^8^Gastroenterology Department, Instituto de Investigación Sanitaria Princesa (IIS‐Princesa), Centro de Investigación Biomédica en Red de Enfermedades Hepáticas y Digestivas (CIBEREHD), Hospital Universitario de La Princesa, Universidad Autónoma de Madrid (UAM), Madrid, Spain, ^9^Gastroenterology, Samsung Medical Center, Seoul, South Korea, ^10^IBD Unit, IRCCS Azienda Ospedaliero‐Universitaria di Bologna, Bologna, Italy

**Contact E‐Mail Address:** m.devillers@erasmusmc.nl

**Introduction:** Side effects, patient preference, and high costs may prompt cessation of anti‐TNF therapy in ulcerative colitis (UC) patients in sustained remission. Given the risk of relapse, careful patient selection is essential. This study aimed to assess relapse rates and identify predictors of relapse following anti‐TNF cessation in UC patients in remission.

**Aims & Methods:** A systematic search on cohort studies reporting relapse rates after cessation of anti‐TNF therapy in ≥ 30 UC patients in clinical remission was performed 6 September 2024. Individual patient data was requested. We excluded patients with steroid use, ≤ 6 months anti‐TNF therapy, ≤ 16 years, previous colectomy (*n* = 4), golimumab as anti‐TNF therapy (*n* = 3) or without follow‐up. Continuation of treatment with immunomodulators or mesalamine at cessation of anti‐TNF was allowed. Relapse was defined as the need to (re)start treatment with steroids, anti‐TNF or other advanced therapies, or the need for surgical treatment, as applied by the original author. Pooled relapse rates were computed using a random‐effects model. A prediction model was built with a Cox proportional hazards model, performance assessed by iteratively leaving one study out (internal‐external cross‐validation, IECV). Missing covariate data were multiply imputed 100 times using the MICE algorithm. Variable selection was guided by three criteria: ≤ 20% missing data, a univariate *p*‐value ≤ 0.2, and input from clinical experts.

**Results:** In total, 654 patients from 8 studies were included in the individual participant data meta‐analysis. At anti‐TNF cessation, median age was 40 years (IQR 31–51) with a median anti‐TNF treatment of 18 months (IQR 18–36). The majority of patients were treated with infliximab (*n* = 610, 93%). Only 42 patients (9%) received a second‐line anti‐TNF therapy. The pooled relapse rate were 24% [95% CI 19%–29%, I^2^ = 44%] and 34% [95% CI 29%–40%, I^2^ = 39] at 1 and 2 years after cessation, respectively. Upon cessation, 211 patients (33%) continued mesalamine, 239 (39%) thiopurines, and 136 (22%) a combination of both. In our model, thiopurine therapy and older age at cessation were associated with a reduced risk of relapse, whereas immunomodulator refractoriness as an indication for starting anti‐TNF therapy was associated with an increased risk. Other factors included in the prediction model were disease extent, type of anti‐TNF therapy, history of anti‐TNF therapy, fecal calprotectin level and endoscopic activity, leading to a *c* statistic of 0.55 [95% CI 0.51–0.59] (Table 1).

**TABLE 1**Predictors of relapse in a pooled Cox regression analysis.

**Table:** 

Variable	Hazard rate	95% confidence interval
Age at discontinuation, per 10 years	0.80	0.72–0.88
Ulcerative colitis disease extent		
Proctitis versus extensive	1.4	0.92–2.1
Left‐sided versus extensive	1.1	0.87–1.5
Type of anti‐TNF therapy, adalimumab versus infliximab	0.91	0.55–1.5
≥ 1 previous anti‐TNF, yes versus no	1.5	0.95–2.3
Indication anti‐TNF therapy immunomodulator refractoriness, yes versus. no/unknown	1.5	1.1–2.2
Concomitant treatment after anti‐TNF therapy		
None versus thiopurine	1.8	1.1–3.0
Mesalamine versus thiopurine	1.2	0.86–1.6
Combination of mesalamine and thiopurines versus thiopurine	1.2	0.81–1.7
Fecal calprotectin (μg/g), per 100	1.1	0.95–1.3
Endoscopic activity, yes (mild, moderate or severe) versus no	1.3	0.91–2.0

**Conclusion:** Approximately 1 in 4 patients experience a relapse of UC within 1 year after anti‐TNF cessation, increasing to 1 in 3 within 2 years. Continuation of thiopurines is associated with a reduced risk. A prediction model for relapse was developed in this large IPD meta‐analysis cohort and may aid in shared decision‐making on anti‐TNF cessation. However, its limited discriminative ability underscores the need for further refinement, incorporating data on microscopic remission and molecular and immunological markers to better capture deeper disease control.

**Disclosure:** Nothing to disclose.

## LB05 | Up‐Front Vedolizumab Versus Conventional Treatment for Checkpoint Inhibitor Induced Colitis—Veico: An Open Label Randomized Clinical Trial

E. K. Dahl ^1^, J. T. Bjerrum^2^, K. Risager Christensen^2^, A. B. Wozniak^2^, J. Fremberg Ilvemark^2^, M. M. Koch^3^, P. Blanche^3^, N. D. Vad^4^, M. Donia^5^, I. M. Svane^5^, J. B. Seidelin^2^

^1^Digestive Diseases, Transplantation and General Surgery, Rigshospitalet, Copenhagen University Hospital, Copenhagen, Denmark, ^2^Gastroenterology, Copenhagen University Hospital, Herlev, Denmark, ^3^Biostatistics, Copenhagen University, Copenhagen, Denmark, ^4^Gastroenterology, Copenhagen University Hospital, North Zealand, Denmark, ^5^Oncology, National Center for Cancer Immune Therapy (CCIT‐DK), Copenhagen University Hospital, Herlev, Denmark

**Contact E‐Mail Address:** emilie.kristine.dahl@regionh.dk

**Introduction:** Immune checkpoint inhibitor (ICI) mediated enterocolitis (IMC) may lead to treatment discontinuation. Corticosteroids for IMC is associated with serious side effects and might affect cancer progression in addition to preclude continued ICI treatment. We hypothesized that vedolizumab, a gut selective α4β7 integrin inhibitor, is effective as a first‐line treatment and investigated whether vedolizumab reduces corticosteroid exposure and facilitate ICI resumption.

**Aims & Methods:** We conducted a single center, open‐label, explorative Phase 2 trial including patients treated with ICI who developed IMC. Patients were randomized 1:1 ratio to receive vedolizumab (300 mg) or standard treatment with corticosteroids, with infliximab used as recue therapy for corticosteroid failure. The primary outcome was cumulative corticosteroid dose at week 30. Key secondary outcomes included cumulative corticosteroid dose at week 10, corticosteroid free remission at week 10 and ICI resumption rates.

**Results:** Twenty‐two patients received vedolizumab, and 19 patients received standard treatment. At week 10, the mean cumulative corticosteroid use was significantly lower in the vedolizumab arm (1157 vs. 2207 mg). By week 30, the mean corticosteroid use remained lower but not statistically significant (1378 vs. 2390 mg, estimated difference 1012 mg, 95% CI ‐229 to 2253, *p* = 0.107), Among patients with less severe IMC, who did not need corticosteroids during screening, vedolizumab‐treated patients used in average 1729 mg less corticosteroid (95% CI 631–2827). Corticosteroid‐free remission at weeks 2 and 10 was more common with vedolizumab. Seven patients (33.3%) in the vedolizumab group resumed with ICI compared to three (16.7%) in the standard arm. In patients with severe colitis, who required upfront corticosteroid treatment, a trend toward longer hospitalization in the vedolizumab arm was observed.

**Conclusion:** For patients with less severe colitis, not needing corticosteroids during screening, vedolizumab may be a viable corticosteroid‐sparing option, enabling faster remission and ICI resumption.

**Disclosure:** The study was funded by Aage og Johanne Louis‐Hansens Fond, National Research grants. Takeda Pharma provided the study drug vedolizumab.

## Hot off the press: IBD treatment 10:00‐11:00/Room Helsinki

## LB06 | Efficacy of Obefazimod in Abtect Phase 3 Induction Trials: Results of 8‐Week Therapy in Subsets of Patients With and Without Prior Inadequate Response to Advanced Therapies

S. Danese ^1^, B. E. Sands^2^, L. Peyrin‐Biroulet^3^, M. C. Dubinsky^4^, B. Siegmund^5^, R. Atreya^6^, T. Hisamatsu^7^, H. Tilg^8^, A. Armuzzi^9^, X. Treton^10^, F. Baert^11^, U. Seidler^12^, F. Cataldi^13^, D. Jacobstein^13^, C. Rabbat^13^, K. Shan^13^, G. DuVall^14^, P. S. Dulai^15^, D. T. Rubin^16^, S. Vermeire^17^

^1^Gastrointestinal Immunopathology, IRCCS San Raffaele Scientific Institute, Vita‐Salute San Raffaele University, Milan, Italy, ^2^Division of Gastroenterology, Icahn School of Medicine at Mount Sinai, New York, New York, USA, ^3^Gastroenterology, INFINY Institute, INSERM NGERE, Nancy University Hospital, Vandoeuvre‐les‐Nancy, France, ^4^Mount Sinai Kravis Children's Hospital, Pediatric GI and Nutrition, New York, New York, USA, ^5^Med. Klinik m.S. Gastroenterologie, Infektiologie und Rheumatologie, Charité ‐ Universitätsmedizin Berlin, Berlin, Germany, ^6^Medical Department 1, University Erlangen‐Nuremberg, Erlangen, Germany, ^7^Kyorin University School of Medicine, Tokyo, Japan, ^8^Department of Medicine, Innsbruck Medical University, Innsbruck, Austria, ^9^Gastroenterology, IBD Center, IRCCS Humanitas Research Hospital, Milan, Italy, ^10^Groupe Hospitalier Prive Ambroise Pare – Hartmann, Institut des MICI, Neuilly sur Seine, France, ^11^Department of Gastroenterology, Az Delta, Roeselare, Belgium, ^12^Medizinische Hochschule Klinik f. Gastroenterlogie, Hannover, Germany, ^13^Abivax, Paris, France, ^14^Tyler Research Institute, Tyler, Texas, USA, ^15^Gastroenterology & Hepatology, Feinberg School of Medicine, Northwestern University, Chicago, Illinois, USA, ^16^University of Chicago Medicine, Chicago, Illinois, USA, ^17^Department of Gastroenterology, University Hospital Leuven, Leuven, Belgium

**Contact E‐Mail Address:** danese.silvio@hsr.it

**Introduction:** Obefazimod (Obe), an oral, once‐daily (QD), small molecule which enhances expression of microRNA‐124 was studied in patients (pts) with moderately to severely active ulcerative colitis (UC) in Phase 2 studies [1–3]. Here we present efficacy endpoints at week 8 for pts with and without prior inadequate response to advanced therapies (AT‐IR‐Yes, AT‐IR‐No) from ABTECT‐1 [NCT05507203] and ABTECT‐2 [NCT05507216] Phase 3 trials.

**Aims & Methods:** The multicenter, randomized, double‐blind, placebo‐controlled ABTECT trials enrolled pts with moderate‐to‐severe UC (modified Mayo score, MMS ≥ 5, with rectal bleeding sub‐score (RBS) ≥ 1 and centrally read endoscopic score ≥ 2) who had inadequate response, loss of response, or intolerance to at least one prior therapy (no upper limit), including corticosteroids, immunosuppressants, biologics, S1P receptor modulators and/or JAK inhibitors (JAKi). Pts were randomized 2:1:1 to Obe 50 mg QD (Obe‐50), Obe 25 mg QD (Obe‐25) or placebo (PBO) for 8 weeks. Clinical remission per MMS and other efficacy measures (clinical response, endoscopic improvement, symptomatic remission, histo‐endoscopic mucosal improvement) were analyzed among AT‐IR‐Yes and AT‐IR‐No pts; all *p*‐values are nominal.

**Results:** Among pts who participated in ABTECT‐1, ABTECT‐2, and the pooled analysis, 45.3% (288/636), 49.4% (314/636), and 47.3% (602/1272) were AT‐IR‐Yes, respectively; 54.7% (348/636), 50.6% (322/636), 52.7% (670/1272) were AT‐IR‐No. In ABTECT‐1, ABTECT‐2, and pooled, a higher proportion of the AT‐IR‐Yes subgroup receiving Obe‐50 achieved all efficacy measures relative to PBO including clinical remission (Obe‐50‐PBO difference: ABTECT‐1 12.7%, *p* = 0.0039; ABTECT‐2 7.6%, *p* = 0.0652, pooled 10.1%, *p* = 0.0009). In the AT‐IR‐Yes subgroup, the difference from PBO across all efficacy measures was lower for pts receiving Obe‐25 relative to Obe‐50 (Table 1). In the AT‐IR‐No subgroup, larger proportions achieved all efficacy measures relative to PBO including clinical remission (Obe‐50‐PBO difference: ABTECT‐1 25.0%, *p* < 0.0001; ABTECT‐2 19.4%, *p* = 0.0004; pooled 22.4%, *p* < 0.0001). In the AT‐IR‐No subgroup, Obe‐50 and Obe‐25 performed similarly in the pooled analysis across efficacy measures. In the subset of pts who failed JAKi, higher proportion of pts receiving Obe‐50 or Obe‐25 achieved clinical response versus PBO. The difference from PBO in clinical response for Obe‐25 and Obe‐50 was similar across lines of treatment, from AT‐IR‐No through 4+ AT‐IRs (Table). Obe was well tolerated with a safety profile similar to previous studies.

**TABLE 1**Efficacy of obefazimod at week 8 in pts with and without prior inadequate response* to AT– ABTECT‐1, ABTECT‐2 and pooled studies.

**Table:** 

	ABTECT‐1^a^ AT‐IR‐Yes			ABTECT‐1^a^ AT‐IR‐No			ABTECT‐2^a^ AT‐IR‐Yes			ABTECT‐2^a^ AT‐IR‐No			Pooled ABTECT trials^a^ AT‐IR‐Yes			Pooled ABTECT trials^a^ AT‐IR‐No		
Efficacy endpoints at week 8, % (*n*)	PBO (*N* = 69)	Obe‐25 (*N* = 70)	Obe‐50 (*N* = 149)	PBO (*N* = 89)	Obe‐25 (*N* = 90)	Obe‐50 (*N* = 169)	PBO (*N* = 79)	Obe‐25 (*N* = 76)	Obe‐50 (*N* = 159)	PBO (*N* = 80)	Obe‐25 (*N* = 83)	Obe‐50 (*N* = 159)	PBO (*N* = 148)	Obe‐25 (*N* = 146)	Obe‐50 (*N* = 308)	PBO (*N* = 169)	Obe‐25 (*N* = 173)	Obe‐50 (*N* = 328)
Clinical remission Placebo adjusted Δ% *p*‐value	1.4 (1)	8.6 (6) 7.1 *p* = 0.0556	14.1 (21) 12.7 *p* = 0.0039	3.4 (3)	35.6 (32) 32.4 *p* < 0.0001	28.4 (48) 25.0 *p* < 0.0001	5.1 (4)	5.3 (4) 0.7 *p* = 0.8529	12.6 (20) 7.6 *p* = 0.0652	7.5 (6)	16.9 (14) 9.5 *p* = 0.0659	27.0 (43) 19.4 *p* = 0.0004	3.4 (5)	6.8 (10) 3.5 *p* = 0.1728	13.3 (41) 10.1 *p* = 0.0009	5.3 (9)	26.6 (46) 21.3 *p* < 0.0001	27.7 (91) 22.4 *p* < 0.0001
Endoscopic improvement Placebo adjusted Δ% *p*‐value	4.3 (3)	14.3 (10) 9.9 *p* = 0.0457	22.1 (33) 17.8 *p* = 0.0011	6.7 (6)	55.6 (50) 48.9 *p* < 0.0001	43.2 (73) 36.4 *p* < 0.0001	8.9 (7)	11.8 (9) 3.5 *p* = 0.4675	26.4 (42) 17.7 *p* = 0.0015	11.3 (9)	31.3 (26) 20.3 *p* = 0.0016	44.7 (71) 33.1 *p* < 0.0001	6.8 (10)	13.0 (19) 6.4 *p* = 0.0687	24.4 (75) 17.8 *p* < 0.0001	8.9 (15)	43.9 (76) 34.9 *p* < 0.0001	43.9 (144) 34.9 *p* < 0.0001
Symptomatic remission Placebo adjusted Δ% *p*‐value	10.1 (7)	27.1 (19) 17.0 *p* = 0.0108	34.9 (52) 25.0 *p* = 0.0001	23.6 (21)	54.4 (49) 31.0 *p* < 0.0001	46.7 (79) 23.2 *p* = 0.0003	15.2 (12)	26.3 (20) 11.3 *p* = 0.0841	37.1 (59) 21.7 *p* = 0.0006	28.8 (23)	39.8 (33) 11.0 *p* = 0.1430	43.4 (69) 14.2 *p* = 0.0329	12.8 (19)	26.7 (39) 13.8 *p* = 0.0031	36.0 (111) 23.4 *p* < 0.0001	26.0 (44)	47.4 (82) 21.3 *p* < 0.0001	45.1 (148) 19.2 *p* < 0.0001
HEMI Placebo adjusted Δ% *p*‐value	1.4 (1)	5.7 (4) 4.3 *p* = 0.1803	12.8 (19) 11.3 *p* = 0.0073	4.5 (4)	37.8 (34) 33.4 *p* < 0.0001	32.0 (54) 27.5 *p* < 0.0001	6.3 (5)	7.9 (6) 1.8 *p* = 0.6596	17.0 (27) 11.0 *p* = 0.0197	8.8 (7)	18.1 (15) 9.3 *p* = 0.0805	30.8 (49) 21.8 *p* = 0.0002	4.1 (6)	6.8 (10) 2.9 *p* = 0.2785	14.9 (46) 11.1 *p* = 0.0005	6.5 (11)	28.3 (49) 21.7 *p* < 0.0001	31.4 (103) 24.7 *p* < 0.0001
Clinical response Placebo adjusted Δ% *p*‐value	21.7 (15)	52.9 (37) 31.2 *p* = 0.0002	54.4 (81) 32.5 *p* < 0.0001	33.7 (30)	75.6 (68) 41.8 *p* < 0.0001	66.9 (113) 33.2 *p* < 0.0001	20.3 (16)	40.8 (31) 21.0 *p* = 0.0043	57.2 (91) 36.8 *p* < 0.0001	46.3 (37)	65.1 (54) 18.8 *p* = 0.0165	69.2 (110) 22.2 *p* = 0.0008	20.9 (31)	46.6 (68) 25.6 *p* < 0.0001	55.8 (172) 34.9 *p* < 0.0001	39.6 (67)	70.5 (122) 30.7 *p* < 0.0001	68.0 (223) 28.2 *p* < 0.0001
										Clinical response, % *AT‐IR pts w/JAK failure* (*n*/*N*1**) Placebo adjusted Δ% *p*‐value *AT‐IR pts w/1 AT failure* (*n*/*N*2**) Placebo adjusted Δ% *p*‐value *AT‐IR pts w/2 AT failures* (*n*/*N*2**) Placebo adjusted Δ% *p*‐value *AT‐IR pts w/3 AT failures* (*n*/*N*2**) Placebo adjusted Δ% *p*‐value *AT‐IR pts w/4+ AT failures* (*n*/*N*2**) Placebo adjusted Δ% *p*‐value			20.0 (7/35) 24.2 (15/62) 20.6 (7/34) 14.3 (4/28) 20.8 (5/24)	47.1 (16/34) 27.0 *p* = 0.0196 53.3 (24/45) 30.5 *p* = 0.0018 48.9 (22/45) 28.6 *p* = 0.0114 42.4 (14/33) 27.3 *p* = 0.0297 34.8 (8/23) 14.4 *p* = 0.2968	54.5 (30/55) 34.4 *p* = 0.0017 50.7 (76/150) 25.3 *p* = 0.0008 67.2 (43/64) 45.5 *p* < 0.0001 61.5 (32/52) 46.3 *p* < 0.0001 50.0 (21/42) 28.7 *p* = 0.0242			

NRI is used for subjects with missing outcome at week 8 and subjects reporting any IE prior to week 8. % Difference is for Obe minus placebo and is based on estimated common risk difference using the Mantel‐Haenszel weights adjusting for the randomization stratification factors: inadequate response to advanced therapies (yes/no), baseline oral corticosteroids usage (yes/no), and region (Japan/rest of world) [ABTECT‐2 only]. *p*‐values are two sided. ^a^: all *p*‐values are nominal. Clinical remission: stool frequency sub‐score (SFS) ≤ 1, rectal bleeding sub‐score (RBS) = 0 and endoscopic sub‐score ≤ 1; Clinical response: decrease from baseline in the MMS ≥ 2 points and ≥ 30% from baseline, plus a decrease in RBS ≥ 1 or an absolute RBS ≤ 1; Endoscopic improvement: endoscopic sub‐score ≤ 1; Symptomatic remission: RBS = 0, SFS < 1; Histo‐endoscopic mucosal improvement (HEMI): combination of histologic improvement (Geboes histologic score ≤ 3.1) and endoscopic improvement (endoscopy sub‐score ≤ 1). *: inadequate response = lack of response, loss of response, or intolerance to advanced therapies. **: *N*1, *N*2 = total number of pts in the respective subset; JAKi: Janus kinase inhibitor.

**Conclusion:** Among AT‐IR‐Yes and AT‐IR‐No subgroups, improvements in clinical, endoscopic, symptomatic and combined endoscopic‐histologic measures were observed in pts treated with Obe at week 8 relative to PBO in ABTECT‐1, ABTECT‐2, and in the pooled analysis. Improvements versus PBO in clinical response were observed in a pooled analysis in AT‐IR‐Yes JAKi‐IR pts and across lines of therapy up to 4+ AT‐IR.

**References**

1. S. Vermeire, V. Solitano, L. Peyrin‐Biroulet, et al., “Obefazimod: A First‐in‐Class Drug for the Treatment of Ulcerative Colitis,” *Journal of Crohn's and Colitis* 17, no. 10 (2023): 1689–1697, 10.1093/ecco‐jcc/jjad067.
2. S. Vermeire, B. E. Sands, H. Tilg, et al., “ABX464 (Obefazimod) for Moderate‐to‐Severe, Active Ulcerative Colitis: A Phase 2b, Double‐Blind, Randomised, Placebo‐Controlled Induction Trial and 48 Week, Open‐Label Extension,” Lancet Gastroenterology & Hepatology 7, no. 11 (2022): 1024–1035, 10.1016/S2468‐1253(22)00233‐3.
3. S. Vermeire, J. Nitcheu, P. Gineste, et al., “Obefazimod in Patients With Moderate‐to‐Severely Active Ulcerative Colitis: Efficacy and Safety Analysis From the 96‐Week Open‐Label Maintenance Phase 2b Study,” *Journal of Crohn's and Colitis* 19, no. 5 (2025): jjaf074, 10.1093/ecco‐jcc/jjaf074.

**Disclosure: Authors reports following potential conflicts of interest:**

**BES:** Abbvie, Abivax, Adiso Therapeutics, Agomab, Alimentiv, Amgen, AnaptysBio, Arena Pharmaceuticals, Artugen Therapeutics, Astra Zeneca, Biolojic Design, Biora Therapeutics, Boehringer Ingelheim, Bristol Myers Squibb, Celltrion, Equillium, Enthera, Enveda Biosciences, Evommune, Ferring, Fzata, Galapagos, Genentech (Roche), Gilead Sciences, GlaxoSmithKline, Gossamer Bio, Imhotex, Index Pharmaceuticals, Innovation Pharmaceuticals, Kaleido, Kallyope, Lilly, Merck & Co., Microba, Microbiotica, Mitsubishi Tanabe, Mobius Care, Morphic Therapeutics, MRM Health, Nexus Therapeutics, Nimbus Discovery, Odyssey Therapeutics, OSE Immunotherapeutics, Janssen, Palisade Bio, Pfizer, Protagonist Therapeutics, Q32 Bio, Rasayana Therapeutics, Recludix Therapeutics, Reistone Biopharma, Sanofi, Sorriso Therapeutics, Spyre Therapeutics, Takeda, Target RWE,Teva, TLL Pharmaceutical, Tr1X, Trex Bio, Union Therapeutics, Ventyx Biosciences.

**SV:** AbbVie, Abivax, AbolerIS Pharma, AgomAb, Alimentiv, Arena Pharmaceuticals, AstraZeneca, Avaxia, BMS, Boehringer Ingelheim, Celgene, CVasThera, Cytoki Pharma, Dr Falk Pharma, Ferring, Galapagos, Genentech‐Roche, Gilead, GSK, Hospira, Imidomics, Janssen, J&J, Lilly, Materia Prima, MiroBio, Morphic, MrMHealth, Mundipharma, MSD, Pfizer, Prodigest, Progenity, Prometheus, Robarts Clinical Trials, Second Genome, Shire, Surrozen, Takeda, Theravance, Tillots Pharma AG, Zealand Pharma.

**LPB:** Abbvie, Abivax, Adacyte, Alimentiv, Amgen, Applied Molecular Transport, Arena, Banook, Biogen, BMS, Celltrion, Connect Biopharm, Cytoki Pharma, Enthera, Ferring, Fresenius Kabi, Galapagos, Genentech, Gilead, Gossamer Bio, GSK, IAC Image Analysis, Index Pharmaceuticals, Inotrem, Janssen, Lilly, Medac, Mopac, Morphic, MSD, Nordic Pharma, Novartis, Oncodesign Precision Medicine, ONO Pharma, OSE Immunotherapeuthics, Pandion Therapeuthics, Par' Immune, Pfizer, Prometheus, Protagonist, Roche, Samsung, Sandoz, Sanofi, Satisfay, Takeda, Telavant, Theravance, Thermo Fischer, Tigenix, Tillots, Viatris, Vectivbio, Ventyx, Ysopia.

**SD:** AbbVie, Ferring, Hospira, Johnson & Johnson, Merck, MSD, Takeda, Mundipharma, Pfizer Inc., Tigenix, UCB Pharma, Vifor, Biogen, Celgene, Allergan, Celltrion, Sandoz, Boehringer Ingelheim.

**PSD:** Abbvie, Abivax, Adiso, Alimentiv, Bristol Meyer Squibb, Celltrion, Genentech, Geneoscopy, Janssen, Pfizer, Takeda.

**MD:** Abbvie, Abivax, Arena Pharmaceuticals, Astra Zeneca, Boehringer Ingelheim International GmbH, Bristol‐Meyer Squibb, Eli Lilly and Company, F. Hoffmann‐La Roche Ltd, Genentech Inc., Gilead, Janssen Pharmaceuticals, Merck, Pfizer Inc., Prometheus Biosciences, Takeda Pharmaceuticals.

**HT:** Abbvie, Abivax, Dr Falk Pharma, ferring, Galapagos, Microbiotica, MSD, Pfizer, Takeda.

**BS:** AbbVie, Abivax, Boehringer Ingelheim, Bristol Myers Squibb, Dr. Falk Pharma, Eli Lilly, Endpoint Health, Falk, Galapagos, Gilead, Janssen, Landos, Materia Prima, PredictImmune, Pfizer, and Takeda; AlfaSigma, CED Service GmbH, MSD, Ferring, Galapagos, Tr1x bio.

**TH:** Mitsubishi Tanabe Pharma Corporation, EA pharma Co. Ltd., AbbVie GK, JIMRO Co. Ltd., Zeria Pharmaceutical Co. Ltd., Kyorin Pharmaceutical Co. Ltd., Nippon Kayaku Co. Ltd., Takeda Pharmaceutical Co. Ltd., Pfizer Inc., Mochida Pharmaceutical Co. Ltd., Boston Scientific Corporation, Kissei Pharmaceutical Co. Ltd., Janssen Pharmaceutical K.K., Pfizer Inc., Eli Lilly, Gilead Sciences, Bristol Myers Squibb, Abivax.

**XT:** Celltrion, AbbVie, Johnson & Johnson, Lilly, Takeda, Alpha Sigma, Dr. Falk, Abivax, Biogen, Fresenius Kabi, MSD, Pfizer, Tillotts, and Thabor Therapeutics.

**RA:** AbbVie, Abivax, AstraZeneca, Bristol‐Myers Squibb, Celltrion Healthcare, Galapagos, Johnson&Johnson, Lilly, MSD, Pfizer, and Takeda Pharma.

**US:** AbbVie, Abivax, Amgen, Galapagos, Janssen, Eli Lilly, Boehringer Ingelheim, Bristol Myers Squibb, Celgene, Gilead Sciences, Pfizer, Roche, Takeda Pharmaceuticals.

**AA:** AbbVie, Abivax, AG Pharma, Alfa Sigma, Astra Zeneca, Biogen, Boehringer Ingelheim, Bristol‐Myers Squibb, Celltrion, Eli‐Lilly, Enthera, Ferring, Galapagos, Gilead, Giuliani, Janssen, Lionhealth, MSD, Nestlé, Novartis, Pfizer, Protagonist Therapeutics, Roche, Samsung Bioepis, Sanofi, Sandoz, Takeda, Teva Pharmaceuticals, Tillots Pharma.

**FB:** AbbVie, Amgen, Eurogenerics, J&J, Arena Pharmaceuticals, Celltrion, Ferring, Galapagos, Janssen, Merck Sharp & Dohme, Pfizer Inc., Takeda, Celgene, Fresenius Kabi, Sandoz.

**DTR:** Abbvie, Abivax SA, Altrubio, Athos Therapeutics Inc., Bristol‐Myers Squibb, Celltrion, Connect BioPharma, Eli Lilly & Co., Genentech (Roche) Inc., Iterative Health, Janssen Pharmaceuticals, Johnson & Johnson, Merck & Co., Odyssey Therapeutics, Pfizer, Sanofi, Spyre, Takeda Pharmaceuticals, Vedanta Biosciences, and Ventyx.

## LB07 | Oral Mufemilast (a Novel Selective Pde4inhibitor) Demonstrates Efficacy and Safety in a Phase II Multicenter, Randomized, Double‐Blind, Placebo‐Controlled Induction Trial in Chinese Patients With Moderate to Severe Ulcerative Colitis

C. Jones ^1^, S. Zhang^2^, C. Zheng^3^, B. R. Liu^4^, X. Zuo^5^, S. Ding^6^, X. Ding^7^, L. Li^8^, Y. Chen^9^, X. Ren^10^, C. Liu^11^, X. Liu^12^, C. Wang^13^, H. Guo^14^, R. Yu^15^, B. Xu^16^, F. Tian^17^, X. Hou^18^, H. Yang^19^, Z. Yu^20^, X. Hu^21^, Y. Zhen^1^, Z. Wang^1^, H. Hai^1^, A. Huo^1^, H. Zhang^1^

^1^Tianjin Hemay Pharmaceuticals, Tianjin, China, ^2^Beijing Friendship Hospital, Beijing, China, ^3^Shengjing Hospital of China Medical University, Medical University, Shenyang, China, ^4^The First Affiliated Hospital of Zhengzhou University, Digestive Internal Medicine, Zhengzhou, China, ^5^Qilu Hospital of Shandong University, Jinan, China, ^6^Gastroenterology, Peking University Third Hospital, Beijing, China, ^7^The First Affiliated Hospital of Ningbo University, Ningbo, China, ^8^Sichuan Provincial People's Hospital, Chengdu, China, ^9^The 1st Affiliated Hospital of Nanchang University, Nanchang, China, ^10^Heilongjiang Provincial Hospital, Harbin, China, ^11^Affiliated Hospital of Binzhou Medical College, Binzhou, China, ^12^Department of Gastroenterology, Xiangya Hospital, Changsha, China, ^13^Digestive, The First Affiliated Hospital of Fujian Medical University, Fuzhou, China, ^14^Chongqing People's Hospital, Chongqing, China, ^15^Chifeng City Hospital, Chifeng, China, ^16^Gastroenterology, Beijing Luhe Hospital Affiliated to Capital Medical University, Beijing, China, ^17^Gastroenterology, Shengjing Hospital of China Medical University, Shenyang, China, ^18^Union Hospital Affiliated to Tongji Medical College of Huazhong University of Science and Technology, Wuhan, China, ^19^The Second Affiliated Hospital of Guangzhou Medical University, Guangzhou, China, ^20^The First Affiliated Hospital of Zhejiang University School of Medicine, Hangzhou, China, ^21^The Second Affiliated Hospital of Anhui Medical University, Hefei, China

**Contact E‐Mail Address:** jonesch3@gmail.com

**Introduction:** Despite recent advances in IBD therapy, there is a need for novel effective, safe oral drugs. Mufemilast has demonstrated sustained efficacy and safety in plaque psoriasis [1]. Here we report the efficacy and safety of Mufemilast at 12 weeks from this Phase 2 trial (NCT05486104).

**Aims & Methods:** Patients with moderate to severe UC were randomized (1:1:1) to placebo (*n* = 31), mufemilast 45 mg (*n* = 31) or 60 mg (*n* = 30) twice daily (BD) for 12 weeks. Eligibility criteria required participants to have a modified Mayo score (mMS) of 4‐9, including an endoscopy score of ≥ 2, inadequate/loss of response to conventional therapy (5‐ASA, Steroids, immunomodulators), or biologics (anti‐TNF, anti‐integrin, anti‐interleukin 12/23). Stable co‐treatment with oral 5′ASAs and corticosteroids was permitted if it was stable for at least ≥ 2 weeks before microscopy within the screening period and needed to remain stable for the duration of the study. Patients with positive Quantiferon tests were also eligible. Efficacy definitions were according to FDA guidance [2].

**Results:** Ninety‐two patients with moderate to severe UC were randomized (1:1:1) placebo (*n* = 31), mufemilast 45 mg (*n* = 31) or 60 mg (*n* = 30) twice daily (BD) for 12 weeks. Efficacy parameters were defined; *clinical remission* as a stool frequency subscore of 0 or 1 and no greater than baseline, a score of 0 for rectal bleeding subscore, and a subscore of 0 or 1 for endoscopy (without friability); *clinical response* as a ≥ 2‐point reduction in mMs from baseline and a ≥ 30% decrease from baseline, with a ≥ 1‐point reduction in rectal bleeding subscore from baseline, or a ≤ 1‐point rectal bleeding subscore; *mucosal healing* a mMS endoscopic subscore of ≤ 1 point^2^. A Chi‐square test was used to compare the differences between groups. Safety evaluated the number and severity of AEs, including vital signs, safety laboratory values, and ECGs. There was 1 SAE (possibly related; thrombophlebitis, a preexisting condition) and 2 AEs that led to discontinuation in the 60 mg group (1 pain in the extremities, 1 headache). Most AEs were mild or moderate, and the number of subjects reporting AEs was evenly distributed across the placebo and mufemilast groups. There were no clinically relevant changes in vital signs, laboratory values, or ECG parameters, which raised any concerns. Table 1 summarizes the efficacy data. A subgroup analysis confirmed similar efficacy and safety in Quantiferon‐positive and negative patients and between Biologic‐naive and Biologic‐exposed patients.

**TABLE 1**(^2^FDA Criteria; FAS) 12 weeks data.

**Table:** 

	Placebo (*n*)	45 mg (*n*)	60 mg (*n*)
Population			
5′ASA concomitant treatment	81%	87%	90%
Moderate (4 ≤ mMS ≤ 6)	45%	52%	50%
Severe (7 ≤ mMS ≤ 9)	55%	48%	50%
Efficacy			
^2^Clinical remission*	13% (4/31)	39% (12/31)	57% (17/30)
^2^Clinical response**	42% (13/31)	87% (27/31)	80% (24/30)
^2^Mucosal healing	16% (5/31)	42% (13/31)	67% (20/30)

*delta versus placebo for clinical remission 44% for 60 mg (*p* < 0.001) and ** 26% for the 45 mg group (*p* < 0.07).

**Conclusion:** Mufemilast efficacy and safety were consistent across subgroups regardless of previous biologic use, suggesting Mufemilast is a viable potential future treatment option in patients with moderate to severe UC that breaks through the current 20% ceiling for clinical remission. A Phase 3 study will be conducted to confirm these results.

**References**

1. C. Jones, J. Zhang, L. Wang, et al., “A Double‐Blind, Placebo‐Parallel Controlled Phase III Clinical Study of the Efficacy and Safety of Hemay005 Tablets in Patients With Moderate to Severe Chronic Plaque Psoriasis in China,” *Arthritis & Rheumatology* 75 (2023): 2208–2210, https://acrabstracts.org/abstract/a-double-blind-placebo-parallel-controlled-phase-iii-clinical-study-of-the-efficacy-and-safety-of-hemay005-tablets-in-patients-with-moderate-to-severe-chronic-plaque-psoriasis-in-china/.
2. Food and Drug Administration. *Ulcerative Colitis: Developing Drugs for Treatment. Guidance for Industry ‐ Draft Guidance* (2022), https://www.fda.gov/media/158016/download.

**Disclosure:** Nothing to disclose.

## LB08 | Discovery of a Novel, Selective Sik3 Inhibitor for the Treatment of Ulcerative Colitis, Crohn'S Diseases and Other Autoimmune Diseases

F. Kolb ^1^, J.‐P. Gallivan^1^, S. De Vos^2^, N. Desroy^2^, D. Moreno^2^, M. Torok^2^, A. Blauw^2^, C. Seemayer^2^, E. Karouzakis^2^, C. Tasset^2^, C. Peixoto^3^, D. Merciris^3^, C. Jagerschmidt^3^, A. Cosson^3^, S. Lavazais^3^, W.‐O. Boucher^2^, P. Raboisson^1^

^1^Onco3R Therapeutics, Leuven, Belgium, ^2^Galapagos NV, Mechelen, Belgium, ^3^Novalix, Romainville, France

**Contact E‐Mail Address:** fabrice.kolb@onco3r.com

**Introduction:** Current treatment options for IBD are limited by low clinical remission rates and loss of response. Most therapies target single pathogenic pathways and while combinations of biologics targeting different pathogenic cytokines can increase clinical remission rates, dosing schedules are inconvenient. JAK inhibitors, target multiple cytokine pathways, but are limited by safety concerns.

Salt‐inducible kinases (SIKs) are a subfamily of serine/threonine kinases in the AMP‐activated protein kinase (AMPK) family and act as key regulators of immune cell signaling. SIK inhibitors have been shown to target multiple cytokine pathways.

Three SIK isoforms exist: SIK1, SIK2 and SIK3. SIK inhibitors targeting multiple isoforms have been investigated in clinical trials. Although there have been signals of activity in patients with ulcerative colitis and psoriasis, limited efficacy and safety concerns related to SIK1 and 2 inhibition have hampered their development.

**Aims & Methods:** We performed structure based drug design to identify highly potent and selective SIK3 inhibitors that could offerbest in class efficacy and safety for the treatment of various auto‐immune diseases. Our effort led to the discovery of 100s of compounds spanning SIK3 binding sites. Compounds were tested in protein and cell‐based assays to evaluate potency and selectivity against SIK3 and in a phenotypic human whole blood TNF*α* assay. Lead optimization involving rigorous testing in safety and pharmacology panels, resulted in the selection of lead candidates that were tested in a variety of translational models to identify the most efficacious compounds. Human PK predictions were performed using non‐clinical in vivo PK assessments and allometric scaling leading to the selection of the drug candidate, which entered GLP toxicology studies.

**Results:** O3R‐5671 is a highly potent and specific SIK3 inhibitor with sub‐nanomolar potency for SIK3 and low activity against SIK1 and 2 and over all human kinases. It demonstrated potent activity in a panel of human primary phenotypic cell assays inhibiting TNF*α*, IL‐12, and IL‐23 release in LPS‐stimulated myeloid cells, and inhibiting TNFa release in LPS‐stimulated human whole blood. O3R‐5671 also inhibited activation of TCR‐stimulated human primary T cells and induced IL‐10 secretion from myeloid cells.

In vivo, O3R‐5671 demonstrated dose‐dependent activity in a mouse LPS challenge model, dose‐dependent efficacy in two distinct mouse models of IBD, and dose‐dependent efficacy in mouse models of IL‐23‐induced psoriasis and psoriatic arthritis. Cross‐species PK and allometric scaling were used to predict O3R‐5671's human PK profile‐ which is expected to be conducive to potent SIK3 inhibition and clinical efficacy in patients with IBD.

O3R‐5671 demonstrated a highly attractive profile in ADME and in vitro safety panels. Six‐week GLP toxicity studies in rats and dogs established no observed adverse effect levels (NOAELs) which support dosing in humans with doses that are predicted to be pharmacologically active with a large safety margin.

**Conclusion:** O3R‐5671 is a highly specific and potent SIK3 inhibitor that is predicted to have a flat PK profile in humans. These properties should allow O3R‐5671 to apply strong drug pressure on SIK3 while sparing SIK1 and 2. This is expected to result in the inhibition of multiple pathogenic cytokines including TNFa and IL‐23, both of which have been validated as IBD targets. A clinical trial is ongoing to establish the safety and PK profile of O3R‐5671 and understand its effect on pharmacodynamic biomarkers.

**Disclosure:** All authors were employed by Galapagos who discovered O3R‐5671 or Novalix, a CRO that was employed by Galapagos. Some authors are employed by Onco3R therapeutics, who are currently developing O3R‐5671.

## LB09 | Mucosal Mrna Expression Profile Identifies Patients With Crohn’S Disease at Risk of Relapse After Anti‐Tnf Withdrawal

M. Devillers ^1^, S. ten Bokkel Huinink^1^, M. Doukas^2^, A. P. Verhaar^1^, C. J. van der Woude^1^, A. de Vries^1^, M. P. Peppelenbosch^1^

^1^Department of Gastroenterology and Hepatology, Erasmus MC University Medical Center, Rotterdam, the Netherlands, ^2^Department of Pathology, Erasmus MC University Medical Center, Rotterdam, the Netherlands

**Contact E‐Mail Address:** m.devillers@erasmusmc.nl

**Introduction:** Anti‐tumor necrosis factor (anti‐TNF) therapy can be safely withdrawn in a subset of patients with long‐standing quiescent Crohn's disease. However, accurately identifying which patients belong to this subset remains challenging. This study aimed to identify mRNA profiles associated with relapse risk and prolonged remission after anti‐TNF withdrawal in CD patients in microscopic remission. Here, we leveraged the NanoString 360 Immuno Panel, which provides significant advantages over bulk sequencing by offering highly multiplexed, sensitive, and automated gene expression analysis of the tumor microenvironment without enzymatic amplification to contrast mucosal immune signatures in patients with and without 3‐year relapse following anti‐TNF withdrawal.

**Aims & Methods:** Patients in the CEASE cohort with mucosal biopsies at anti‐TNF withdrawal who later relapsed were matched to those in sustained remission based on biopsy location (ileum or colon).^1^ For patients with a relapse, only biopsies corresponding to the site of disease activity at relapse were selected for mRNA analysis. Microscopic remission at baseline was confirmed by a blinded expert pathologist. Relapse was defined as endoscopically confirmed inflammation, while sustained remission was defined as no relapse during follow‐up. 785 mRNA's of the nCounter Host Response Profiling panel were measured, and corrected to the expression of household genes (IO 360 panel). The 3 most up‐ and downregulated relapse‐associated mRNAs were identified by first selecting on significance (*p* < 0.05), followed by ranking based on log2 fold change. mRNA levels were normalized to the cohort mean, and the score was calculated as the sum of the most upregulated divided by the sum of the most downregulated mRNA's. Hence, a Cox regression and ROC analysis were performed to examine the relationship between the score and relapse.

**Results:** Eleven patients who relapsed were matched to 11 in sustained remission based on biopsy location (7 colon, 4 ileum). Median age was 40 years, 45% male, with median anti‐TNF exposure of 5.2 years. Baseline characteristics were comparable between groups; of note, 6/11 (55%) in the relapse group versus 4/11 (36%) in sustained remission received concomitant immunomodulators (*p* = 0.67). Median follow‐up was 3.3 years (IQR 2.6) and median time to relapse 15.8 months (IQR 9.5). The score, based on the 3 most up‐ and downregulated mRNAs (IFITM1, PIK3CA, RBPJ, IRF3, THOP1, NEU1), was higher in the relapse group (median 2.94 (IQR 1.2–6.0) versus 0.36 (IQR 0.24–0.56), *p* = 0.001) and associated with relapse risk (HR per one‐point 1.25, 95% CI 1.08–1.45). The AUC was 0.89, with the best cut‐off of 0.98 based on the Youden index, resulting in a sensitivity of 0.83 and specificity of 1.

**TABLE 1**

**Table:** 

mRNA	Mean relapse group	Mean sustained remission group	Log 2 fold change	*p*‐value	Immunological function
IFITM1‐mRNA	5.09E+14	1.88E+14	1.43	0.012	Regulates CD4+ T helper cell differentiation
PIK3CA‐mRNA	3.36E+14	1.28E+14	1.39	0.004	Enhancing CD8+ T‐cell action via increased IL‐2
RBPJ‐mRNA	4.22E+14	1.73E+14	1.28	0.032	Critical regulator of T cell differentiation
NEU1‐mRNA	1.59E+14	3.70E+14	−1.22	0.046	Cleaves sialic acids from monocyte glycoproteins
THOP1‐mRNA	2.31E+14	5.97E+14	−1.37	0.010	THOP1^‐/‐^ mice exhibit better survival in sepsis
IRF3‐mRNA	5.08E+13	1.70E+14	−1.74	0.014	Canonical innate immunity transcription factor

**Conclusion:** This study identifies a specific mucosal mRNA profile associated with relapse risk after anti‐TNF withdrawal in Crohn disease patients in clinical, endoscopic and microscopic remission. This profile reliably distinguishes high‐risk patients in this case‐control study but requires further validation. Furthermore, it provides insights in mucosal immune mechanisms, suggesting that strong innate immunity may protect against CD relapse, whereas adaptive immunity is associated with risk for relapse.

**References**

1. S. Ten Bokkel Huinink, D. C. de Jong, D. Nieboer, et al., “Validation and Update of a Prediction Model for Risk of Relapse After Cessation of Anti‐TNF Treatment in Crohn's Disease,” *European Journal of Gastroenterology & Hepatology* 34, no. 10 (2022): 983–992, 10.1097/MEG.0000000000002403.

**Disclosure:** Nothing to disclose.

## LB10 | Artificial Intelligence Endoscopy Scoring Demonstrates That Tdm‐Based Infliximab Dose‐Intensification is Superior to Standard Dosing in Patients With Acute Severe Ulcerative Colitis: A Post‐Hoc Analysis of the Titrate Study

K. B. Gecse ^1^, J. van Oostrom^1^, S. T. Rietdijk^2^, S. O. Frigstad^3^, G. A. Doherty^4^, P. Irving^5^, D. Laharie^6^, F. de Voogd^1^, M. Pruijt^1^, S. Anjie^1^, L. Oldenburg^1^, A. Volkers^1^, L. Mulders^1^, R. de la Croix‐Vingerling^2^, R. Koppes^2^, M. M. Islam^3^, K. Hammersboen Bjorlykke^7^, K. Haug^7^, C. Coe^4^, D. Mould^8^, J. James^8^, C. Moore^9^, M. Hulshoff^1^, M. Lowenberg^1^, A. Neefjes‐Borst^10^, R. Mathot^11^, E. Clasquin^1^, K. K. Jørgensen^12^, S. Gurm^13^, M. Byrne^13^, G. R. D'Haens^1^

^1^Department of Gastroenterology and Hepatology, Amsterdam University Medical Center, Amsterdam, the Netherlands, ^2^Department of Gastroenterology and Hepatology, Onze Lieven Vrouwen Gasthuis, Amsterdam, the Netherlands, ^3^Department of Gastroenterology and Hepatology, Vestre Viken HF, Gjettum, Norway, ^4^Department of Gastroenterology and Hepatology, School of Medicine, St Vincent's Hospital, University College Dublin, Dublin, Ireland, ^5^Department of Gastroenterology and Hepatology, Guy's and St Thomas' Hospital, London, UK, ^6^Department of Gastroenterology and Hepatology, Centre Hospitalier Universitaire de Bordeaux, Bordeaux, France, ^7^Department of Gastroenterology and Hepatology, Akershus Universitetssykehus, Nordbyhagen, Norway, ^8^Baysient LLC, Fort Meyers, Florida, USA, ^9^BÜHLMANN Laboratories AG, CPO, Basel, Switzerland, ^10^Department of Pathology, Amsterdam University Medical Center, Amsterdam, the Netherlands, ^11^Department of Hospital Pharmacy and Clinical Pharmacology, Amsterdam University Medical Center, Amsterdam, the Netherlands, ^12^Department of Gastroenterology and Hepatology, Faculty of Medicine, Akershus Universitetssykehus, University of Oslo, Nordbyhagen, Norway, ^13^Dova Health Intelligence, Vancouver, British Columbia, Canada

**Contact E‐Mail Address:** krisztina.gecse@gmail.com

**Introduction:** Up to 40% of patients with acute severe ulcerative colitis (ASUC) do not respond to infliximab (IFX) [1] presumably due to insufficient drug exposure [1, 2]. We investigated whether personalized IFX induction using predefined serum concentrations was superior to standard dosing.

**Aims & Methods:** In this prospective, multi‐center trial, adult IFX‐naive steroid‐refractory ASUC patients were randomized 1:1 to standard (SD) or personalized IFX dosing (PD). After an initial 5 mg/kg IFX infusion, SD was 5 mg/kg IFX at week 2 and 6. In PD, additional 5 mg/kg IFX infusions were administered guided by a Bayesian pharmacokinetic algorithm (iDose) aiming at IFX serum concentrations > 28 μg/mL day 0–28 and > 15 μg/mL day 29–42 [3]. The primary composite endpoint was clinical and endoscopic response at day 42 (Lichtiger score < 10 and ≥ 3 points drop and UCEIS ≥ 2 points drop from baseline). Video‐endoscopies were performed at baseline, day 42 and 182 and scored by 2 expert readers (XR) with adjudication by a third XR and post hoc also by the DovaVision UC AI tool (AI‐R) [4]. Key secondary endpoints were day 42 clinical response, day 42 endoscopic response, day 182 clinical remission (Lichtiger ≤ 3), day 182 endoscopic remission (UCEIS ≤ 1 on all components), and safety.

**Results:** Forty‐eight patients were randomized (23 PD/25 SD) and 31 completed the 26‐week study (19 PD/12 SD). Patient characteristics were comparable. Median cumulative IFX dose until day 42 was 18.41 mg/kg [1.77, 20.27] for PD versus 13.79 mg/kg [10.38, 14.82] for SD. The primary composite endpoint at day 42 was not met with XR (57% in PD vs. 44% in SD; *p* = 0.564). However, AI‐R showed a significantly higher composite response rate in PD (74% in PD vs. 32% in SD; *p* = 0.0047). PD showed higher day 42 clinical response versus SD (91% vs. 64%; *p* = 0.039). Day 42 endoscopic response was observed in 57% in PD versus 44% in SD (*p* = 0.564) with XR and 74% in PD versus 32% in SD with AI‐R (*p* = 0.0047). The proportion of patients in clinical and endoscopic remission at day 182 was higher in PD than in SD (65% vs. 36%; *p* = 0.082). Day 182 endoscopic remission with AI‐R was higher in PD (52% in PD and 20% in SD; *p* = 0.0337). SAEs occurred in 9% of patients on PD versus 20% of patients on SD. Following an interim analysis, the trial was discontinued based on futility.

**TABLE 1**Baseline characteristics.

**Table:** 

	Personalized treatment (*n* = 23)	Standard treatment (*n* = 25)
Gender = male (%)	11 (47.8)	15 (60.0)
Age (year) (median [IQR])	37.00 [24.50, 57.50]	42.00 [30.00, 56.00]
Disease duration (years) (median [IQR])	1.75 [0.62, 5.30]	6.16 [2.61, 17.21]
Naive to advanced therapy (%)	18 (78.3)	21 (84.0)
Lichtiger score (median [IQR])	14 [13, 16]	14 [12, 15]
Total Mayo score (median [IQR])	11 [11, 11]	11 [10, 11]
UCEIS (median [IQR])	6 [5, 7]	6 [5, 7]
CRP (mg/L) (median [IQR])	49.00 [35.70, 70.30]	51.00 [17.00, 105.00]
Albumin (g/L) (median [IQR])	26.00 [23.00, 30.50]	31.00 [23.00, 34.00]

Advanced therapy was defined as treatment with any biologics, JAK inhibitor or S1P modulator.

**Abbreviations:** CRP, C‐reactive protein; IQR = interquartile range; UCEIS, Ulcerative Colitis Endoscopic Index of Severity.

**Conclusion:** Personalized IFX dosing was superior to standard dosing in ASUC when endoscopies were read by AI. This is the first IBD study where the primary endpoint, missed by expert assessment, was overturned and met by AI assessment instead. This demonstrates the potential of AI to fundamentally improve clinical trial methodology.

**References**

1. C. H. Seow, A. Newman, S. P. Irwin, A. H. Steinhart, M. S. Silverberg, and G. R. Greenberg, “Trough Serum Infliximab: A Predictive Factor of Clinical Outcome for Infliximab Treatment in Acute Ulcerative Colitis,” *Gut* 59, no. 1 (2010): 49–54, 10.1136/gut.2009.183095.
2. K. Papamichael, T. Van Stappen, N. Vande Casteele, et al., “Infliximab Concentration Thresholds During Induction Therapy Are Associated With Short‐term Mucosal Healing in Patients With Ulcerative Colitis,” *Clinical Gastroenterology and Hepatology* 14, no. 4 (2016): 543–549, 10.1016/j.cgh.2015.11.014.
3. B. Ungar, Y. Mazor, R. Weisshof, et al., “Induction Infliximab Levels Among Patients With Acute Severe Ulcerative Colitis Compared With Patients With Moderately Severe Ulcerative Colitis,” *Alimentary Pharmacology & Therapeutics* 43, no. 12 (2016): 1293–1299, 10.1111/apt.13631.
4. M. Byrne, J. Requa, R. Panaccione, et al., “Development and Validation of a Novel AI‐Based Computer Vision Solution for Ulcerative Colitis Severity Scoring on Video for Real World Using High‐Volume Expert‐Annotated Video Frames,” *United European Gastroenterology Journal* (October 2025).

**Disclosure:** This project received grant support from Pfizer Inc. and research support from Baysient LLC, Buhlmann Laboratories and Dova Health Intelligence.

## Colorectal ESD: The better way? 14:30‐15:30/Room A1

## LB11 | Efficacy of Computer‐Aided Detection System on Adenomatous and Serrated Lesion Detection During Colonoscopy: Results From a Multinational Randomized Controlled Trial in the Asia‐Pacific (Project Cad)

M. Sekiguchi ^1,2^, C.‐Y. Chang^3,4^, K. S. Han^5^, S. Pattarajierapan^6^, K. Sumiyama^7^, L. H. S. Lau^8,9^, H. Kobayashi^10^, R. Lui^11^, C. J. L. Khor^12,13^, K. Shinmura^14^, H.‐M. Chiu^15^, J. Lee^16^, C.‐Y. Kuo^3^, B. Kim^5^, M. Ito^7^, K. Nakamura^1^, Y. Kakugawa^1^, T. Uozumi^17^, Y. Hirai^17^, R. Kawamura^17^, F.‐J. Lee^3^, K. W. Wu^3^, Y.‐T. Chen^3^, T.‐L. Ma^3^, C. W. Hong^5^, B. Kim^5^, D.‐K. Sohn^5^, S. Khomvilai^6^, T. Futakuchi^7^, L. Y. K. Lam^9^, A. J. Hui^9^, N. Chi Fung^9^, A. Fujimoto^10^, T. Matsuda^10^, M. S. Lim^12^, C. S. Clair^18^, T. Shibata^19^, Y. Saito^20^, Project CAD Study Group

^1^Endoscopy Division/Cancer Screening Center, National Cancer Center Hospital, Tokyo, Japan, ^2^Division of Screening Technology, National Cancer Center Institute for Cancer Control, Tokyo, Japan, ^3^Department of Internal Medicine, Fu Jen Catholic University Hospital, New Taipei City, Taiwan, ^4^School of Medicine, College of Medicine, Fu Jen Catholic University, New Taipei City, Taiwan, ^5^Research Institute and Hospital, National Cancer Center, Center for Colorectal Cancer, Center for Cancer Prevention and Detection, Goyang, South Korea, ^6^Surgical Endoscopy Colorectal Division, Department of Surgery, Chulalongkorn University and King Chulalongkorn Memorial Hospital, Bangkok, Thailand, ^7^Department of Endoscopy, The Jikei University School of Medicine, Tokyo, Japan, ^8^Department of Medicine and Therapeutics, Faculty of Medicine/Institute of Digestive Disease/Li Ka Shing Institute of Health Sciences, The Chinese University of Hong Kong, Hong Kong, Hong Kong, ^9^Division of Gastroenterology and Hepatology, CUHK Medical Center, Hong Kong, Hong Kong, ^10^Division of Gastroenterology and Hepatology, Toho University Omori Medical Center, Tokyo, Japan, ^11^Department of Medicine and Therapeutics, Faculty of Medicine/Institute of Digestive Disease, The Chinese University of Hong Kong, Hong Kong, Hong Kong, ^12^Department of Gastroenterology and Hepatology, Singapore General Hospital, Singapore, Singapore, ^13^Duke‐NUS Medical School, Singapore, Singapore, ^14^Department of Gastroenterology and Endoscopy, National Cancer Center Hospital East, Kashiwa, Japan, ^15^Department of Internal Medicine, National Taiwan University Hospital, Taipei, Taiwan, ^16^Division of Gastroenterology and Hepatology, Department of Medicine, Yong Loo Lin School of Medicine, Institute for Health Innovation and Technology (iHealthtech), National University of Singapore, Singapore, Singapore, ^17^Endoscopy Division, National Cancer Center Hospital, Tokyo, Japan, ^18^Department of International Clinical Development, National Cancer Center Hospital, Tokyo, Japan, ^19^Biostatistics Section, Research Management Division, National Cancer Center Hospital, Clinical Research Support Office, Tokyo, Japan, ^20^Endoscopy Division, National Cancer Center Hospital, Tokyo, Japan

**Contact E‐Mail Address:** masekigu@ncc.go.jp

**Introduction:** Computer‐aided detection (CADe) systems have gained attention for their use in colonoscopy, with growing evidence of increased adenoma detection rates (ADR). However, results remain inconsistent, and debate regarding their usefulness continues, including their impact on serrated lesion (SL) detection and performance across varying levels of endoscopist expertise. Given that CADe is used as a real‐time assistive tool, it is crucial to confirm its efficacy in lesion detection before considering long‐term outcomes.

**Aims & Methods:** We conducted a multinational, multicenter randomized controlled trial in the Asia‐Pacific (Project CAD/NCCH2217; jRCT1032230396) to assess the efficacy of CADe in improving lesion detection during colonoscopy performed as part of colorectal cancer (CRC) screening [1]. We enrolled asymptomatic individuals aged 50–79 years undergoing either primary screening colonoscopy or colonoscopy following a positive fecal immunochemical test (FIT), who had not received colonoscopy in the past 5 years. Participants were randomly assigned (1:1) to the CADe group (colonoscopy with CADe assistance) or the standard group (colonoscopy without CADe) using a minimization method to balance age, sex, colonoscopy indication, endoscopist experience, and country/region. In the CADe group, approved CADe devices were used in accordance with each country's regulations, including EndoBRAIN‐EYE, ENDO‐AID, CAD EYE, and WISE VISION. All procedures were performed by endoscopists with experience of ≥ 500 colonoscopies. The primary endpoint was adenoma detection rate (ADR). Secondary endpoints included adenomas per colonoscopy (APC), advanced ADR (AADR), sessile serrated lesion detection rate (SSLDR), sessile serrated lesions per colonoscopy (SSLPC), scope withdrawal time, and the resection rate of non‐neoplastic lesions. Planned sample size was 1400 cases (one‐sided *α* = 0.05, 80% power).

**Results:** Of the 1399 participants enrolled between December 28, 2023, and March 6, 2025, 1373 were included in the full analysis set (688 and 685 in the CADe and standard groups, respectively). In both groups, the proportions of primary screening and post‐FIT colonoscopies were 75% and 25%, respectively. No differences were observed between the CADe and standard groups in ADR (54.7% vs. 53.9%; odds ratio [OR] 1.04; 95% confidence interval [CI], 0.83–1.30, one‐sided *p* = 0.38), APC (1.17 vs. 1.24; risk ratio [RR], 0.94; 95% CI, 0.81–1.09), or AADR (5.5% vs. 7.6%; OR, 0.71; 95% CI, 0.46–1.10). The CADe group showed higher SSLDR (9.0% vs. 6.0%; OR, 1.55; 95% CI, 1.03–2.33) and SSLPC (0.11 vs. 0.07; RR, 1.65; 95% CI, 1.09–2.50). Subgroup analysis based on endoscopist experience demonstrated a higher SSLDR in the CADe group among those with 500–4999 (10.1% vs. 5.4%) and 5000–9999 (11.9% vs. 3.6%) prior colonoscopies, but not among those with experience of ≥ 10,000 procedures (7.0% vs. 7.5%). There was no difference between groups in the non‐neoplastic lesion resection rate (14.7% vs. 13.4%; OR, 1.11; 95% CI, 0.82–1.50), and the median scope withdrawal time in cases without polypectomy was 9 min in both groups.

**Conclusion:** CADe assistance did not improve adenoma detection where high ADR was already achieved with standard colonoscopy. Nevertheless, it enhanced SSL detection, except among highly experienced endoscopists, without prolonging withdrawal time or increasing unnecessary polypectomies. While our findings do not overstate the efficacy of CADe, they support a reasonable and practical role for its use in improving colonoscopy quality in CRC screening.

**References**

1. M. Sekiguchi, K. Shinmura, K. Sumiyama, et al., “Protocol for a Multicenter Randomized Controlled Trial to Assess the Usefulness of Computer‐Aided Detection Systems for Colonoscopy in Colorectal Cancer Screening in the Asia‐Pacific Region (Project CAD/NCCH2217),” *Japanese Journal of Clinical Oncology* 55, no. 7 (2025): 837–840, 10.1093/jjco/hyaf043.

**Disclosure:** The authors have no conflicts of interest related to this study.

## LB12 | Safety and Efficacy of Extended Surveillance Intervals Following Colorectal ESD: An International Multicenter Study

P. Brouzeng ^1^, J. Jacques^2^, J. Albouys^3^, P. Carrier^2^, R. Legros^1^, H. Lepetit^4^, S. Geyl^2^, L. Alfarone^5^, A. Qatomah^6^, C. Hassan^7^, D. Ramai^6^, H. Aihaiara^6^

^1^CHU Limoges, Limoges, France, ^2^Department of Hepato‐Gastro‐Enterology, CHU Limoges, Limoges, France, ^3^Department of Gastroenterology, CHU Limoges, University of Limoges, Limoges, France, ^4^Gastroenterology Unit, CHU Limoges, Limoges, France, ^5^Milan, Italy, ^6^Brigham and Women's Hospital, Massachusetts, Boston, USA, ^7^Gastroenterology, Humanitas University, Milan, Italy

**Contact E‐Mail Address:** brouzengpaul@hotmail.com

**Introduction:** Post‐ESD surveillance guidelines remain heterogeneous, with many centers favoring early surveillance colonoscopy (SC) despite low recurrence rates after curative resection. This study aimed to evaluate the safety and outcomes of delayed SC after R0 colorectal ESD for benign lesions and to identify risk factors for metachronous advanced neoplasia.

**Aims & Methods:** We retrospectively analyzed 1097 patients who underwent colorectal ESD between 2013 and 2021. Clinical, procedural, and histological characteristics were recorded. Among 778 patients with available surveillance data, outcomes were compared between those who had early SC (< 12 months) and those with delayed SC (> 24 months). Rates of recurrence, advanced neoplasia, and colorectal cancer were assessed. Risk factors for metachronous advanced neoplasia were evaluated via univariate and multivariate analysis.

**Results:** Mean patient age was 74.3 ± 10.8 years; 54.1% were male. En bloc and R0 resection rates were 95.8% and 83.3%, respectively. Among patients with surveillance data, 82.6% underwent early SC, and 17.4% had late‐only SC. The rate of advanced adenoma at first SC was low and comparable between early and late groups (3.9% vs. 6.7%, *p* = 0.167). The overall rate of metachronous advanced adenoma was 6.5%, with no significant difference between early and late strategies (6.1% vs. 8.9%, *p* = 0.25). All four colorectal cancers were detected in the early group; none occurred in the late‐only group. Recurrence on the ESD scar was rare (1.5% overall), and no late recurrences occurred after a normal SC1. The only significant predictor of metachronous advanced neoplasia was the number of synchronous lesions at index colonoscopy (OR 1.16, 95% CI 1.07–1.27; *p* < 0.01).

**Conclusion:** Delayed surveillance colonoscopy 3 years after R0 colorectal ESD for benign lesions appears safe and effective in selected patients. The multiplicity of synchronous lesions could guide risk stratification. These findings support extending SC intervals after curative ESD, with potential to reduce unnecessary procedures.

**References**

1. P. Pimentel‐Nunes, D. Libânio, B. A. J. Bastiaansen, et al., “Endoscopic Submucosal Dissection for Superficial Gastrointestinal Lesions: European Society of Gastrointestinal Endoscopy (ESGE) Guideline ‐ Update 2022,” *Endoscopy* 54, no. 6 (June 2022): 591–622, 10.1055/a‐1811‐7025.
2. M. Dai, X. Xiao, C. L. T. Guo, et al., “The Long‐Term Risk of Metachronous Advanced Adenoma Recurrence After Endoscopic Submucosal Dissection for Colorectal Neoplasia: A Propensity‐Score Matched Longitudinal Cohort With 5‐Year Follow‐Up,” *United European Gastroenterology Journal* 13, no. 2 (December 21, 2024): 210–219, 10.1002/ueg2.12735.
3. N. Yoshida, Y. Naito, K. T. H. Siah, et al., “High Incidence of Metachronous Advanced Adenoma and Cancer After Endoscopic Resection of Colon Polyps ≥ 20 mm in Size,” *Digestive Endoscopy* 28, no. 2 (March 2016): 194–202, 10.1111/den.12551.

**Disclosure:** Nothing to disclose.

## LB13 | Transforming Radiation Proctopathy Management: Real‐World Evidence on Purastat From a Uk Teaching Hospital

M. H. Khizar, S. Coda, V. N. Vinoth Nadesalingam

Gastroenterology, Barking, Havering and Redbridge University Hospitals NHS Trust, London, UK

**Contact E‐Mail Address:** muhammad.khizar14@hotmail.com

**Introduction:** Radiation proctopathy (RP) is a common late complication of pelvic radiotherapy, affecting up to 20% of patients treated for pelvic malignancies. It manifests as rectal bleeding, anemia, urgency, and diarrhea, significantly reducing the quality of life. Argon Plasma Coagulation (APC) remains the standard endoscopic therapy but has notable limitations, including high recurrence rates, the need for multiple sessions, and risks such as ulceration and perforation.

PuraStat, a synthetic self‐assembling peptide hydrogel, offers an innovative alternative by creating a transparent matrix that promotes hemostasis and mucosal healing without thermal injury. While small series have shown benefit, robust real‐world evidence is lacking.

**Aims & Methods:** We assessed real‐world outcomes of PuraStat in RP at a UK tertiary center (2023–2024). Consecutive patients with chronic radiation‐induced rectal bleeding were included and treated by a single endoscopist. Patients were stratified into two cohorts:

1. De novo PuraStat—no prior APC (*n* = 50)
2. APC → PuraStat—persistent or recurrent symptoms despite APC (*n* = 50)

PuraStat was applied during flexible sigmoidoscopy, either as monotherapy or following APC. The primary outcome was resolution or improvement of rectal bleeding. Secondary outcomes included the number of repeat endoscopic interventions within 6 months and treatment‐related adverse events.

**Results:** A total of 100 patients were included (median age: 74 years, range 64–85; 52% male). Most had prostate or gynecological cancer treated with pelvic radiotherapy.

— De novo PuraStat (*n* = 50): All achieved symptomatic relief, with marked bleeding reduction and endoscopic improvement. Follow‐up sigmoidoscopy showed mucosal healing or mild residual proctitis in 92%. Mean repeat sessions: 1.3 (range 0–3) over 6 months.
— APC → PuraStat (*n* = 50): These patients had undergone a median of 3 APC sessions with limited success. PuraStat led to complete symptom control in all cases, reducing the need for frequent interventions (mean: 1.5 sessions over 6 months).

No adverse events (e.g., perforation, infection, severe pain) were reported in either group. Patients with severe radiation‐associated vascular ectasia (RAVE) and refractory bleeding demonstrated excellent response to PuraStat.

**Conclusion:** This large real‐world series demonstrates that PuraStat is a safe, effective, and well‐tolerated therapy for radiation proctopathy, whether used as first‐line treatment or following APC failure. It provided rapid and sustained bleeding control, significantly reduced the need for repeat endoscopic interventions, and promoted mucosal healing without the risks associated with thermal therapies. Importantly, PuraStat showed consistent efficacy even in patients with severe radiation‐associated vascular ectasia (RAVE) and those refractory to conventional modalities, highlighting its role as both a frontline and salvage option. These findings position PuraStat as a valuable addition to the therapeutic armamentarium for radiation‐induced rectal bleeding. Larger prospective, multicenter studies and cost‐effectiveness analyses are warranted to validate these results and define its optimal place in treatment algorithms.

**TABLE 1**Treatment outcomes by group.

**Table:** 

Group	*n*	Symptom relief (%)	Mean repeat sessions
De novo PuraStat	50	100	1.3
APC → PuraStat	50	100	1.5

**References**

1. K. White, and C. C. Henson, “Endoscopically Delivered PuraStat for the Treatment of Severe Haemorrhagic Radiation Proctopathy: Service Evaluation of a New Endoscopic Treatment for a Challenging Condition,” *Frontline Gastroenterology* 12, no. 1 (2021): 37–42, 10.1136/flgastro‐2020‐101735.
2. F. T. Van de Wetering, L. Verleye, H. J. N. Andreyev, et al., “Non‐Surgical Interventions for Late Rectal Problems (Proctopathy) of Radiotherapy in People Who Have Received Radiotherapy to the Pelvis,” *Cochrane Database of Systematic Reviews* 4 (2016): CD003455, 10.1002/14651858.CD003455.pub2.
3. H. J. N. Andreyev, S. E. Davidson, C. Gillespie, W. H. Allum, and E. Swarbrick, “Practice Guidance on the Management of Acute and Chronic Gastrointestinal Problems Arising as Result of Treatment for Cancer,” *Gut* 61, no. 2 (2012): 179–192, 10.1136/gutjnl‐2011‐300563.
4. D. S. Dahiya, A. Kichloo, F. Tuma, M. Albosta, and F. Wani, “Radiation Proctitis and Management Strategies,” *Clinical Endoscopy* 55, no. 1 (2022): 22–32, 10.5946/ce.2020.288.
5. T. Rustagi, and H. Mashimo, “Endoscopic Management of Chronic Radiation Proctopathy,” *World Journal of Gastroenterology* 17, no. 41 (2011): 4554–4562, 10.3748/wjg.v17.i41.4554.
6. L. Lenz, R. Rohr, F. Nakao, E. Libera, and A. Ferrari, “Chronic Radiation Proctopathy: A Practical Review of Endoscopic Treatment,” *World Journal of Gastrointestinal Surgery* 8, no. 2 (2016): 151–158, 10.4240/wjgs.v8.i2.151.

**Disclosure:** Nothing to disclose.

## LB14 | Histological Risk Stratification for Lymph Node Metastasis in Pt2 Rectal Cancer After Local Excision: A Multicentre International Study

S. C. Albers ^1^, L. R. Moolenaar^2^, L. M. Moons^3^, M. M. Lacle^4^, P. G. Doornebosch^5^, E. G. Rutten^6^, A. W. van de Ven^7^, P. Drillenburg^8^, D. D. Zimmerman^9^, D. E. Ploeg^10^, A. Spinelli^11^, P. Spaggiari^12^, A. Wolthuis^13^, G. De Hertogh^14^, J. Tuynman^2^, J. Wiggers^2^, E. Dekker^1^, M. M. Lange^15^, R. Hompes^2^, B. A. Bastiaansen^1^

^1^Department of Gastroenterology & Hepatology, Amsterdam University Medical Center, Amsterdam, the Netherlands, ^2^Department of Surgery, Amsterdam University Medical Center, Amsterdam, the Netherlands, ^3^Department of Gastroenterology & Hepatology, University Medical Center Utrecht, Utrecht, the Netherlands, ^4^Department of Pathology, University Medical Center Utrecht, Utrecht, the Netherlands, ^5^Department of Surgery, IJsselland Hospital, Capelle aan de IJssel, the Netherlands, ^6^Department of Pathology, IJsselland Hospital, Capelle aan de IJssel, the Netherlands, ^7^Department of Surgery, Flevo Hospital, Almere, the Netherlands, ^8^Department of Pathology, OLVG, Amsterdam, the Netherlands, ^9^Department of Surgery, Elisabeth‐TweeSteden Hospital, Tilburg, the Netherlands, ^10^Department of Pathology, Elisabeth‐TweeSteden Hospital, Tilburg, the Netherlands, ^11^Department of Surgery, IRCCS Humanitas Research Hospital, Milan, Italy, ^12^Department of Pathology, IRCCS Humanitas Research Hospital, Milan, Italy, ^13^Department of Surgery, University Hospital Leuven, Leuven, Belgium, ^14^Department of Pathology, University Hospital Leuven, Leuven, Belgium, ^15^Department of Pathology, Amsterdam University Medical Center, Amsterdam, the Netherlands

**Contact E‐Mail Address:** s.c.albers@amsterdamumc.nl

**Introduction:** Local excision (LE) is increasingly used to treat early rectal cancer (cT1‐2N0M0), but its role in pT2 cancer remains controversial due to a reported 20%–30% risk of lymph node metastasis (LNM). Since this risk is largely linked to adverse histological features, patients without these risk factors may safely avoid completion total mesorectal excision (cTME). We aimed to refine LNM risk in pT2 rectal cancer following LE to better guide decisions on rectal‐preserving treatment strategies.

**Aims & Methods:** Patients with pT2 rectal cancer who underwent en bloc LE for cT1‐2N0M0 lesions were retrospectivelly enrolled from seven international centers (2015–2025). Eligible patients received either cTME or active surveillance with MRI follow‐up for at least 2 years. Exclusion criteria were positive resection margins (< 0.1 mm), synchronous or recurrent colorectal cancer and (neo)adjuvant therapy. LNM were confirmed through TME histology or considered absent when no suspicious lymph nodes appeared on follow‐up MRI. A detailed histological review of LE specimens was conducted by specialized pathologists at each participating center using a standardized protocol. Assessed risk factors included high‐grade differentiation and/or mucinous subtype, lymphovascular invasion (small and/or large vessels), tumor budding grade 2–3 and/or poorly differentiated clusters grade 2–3, and perineural invasion. Primary endpoint was LNM rate in patients without histological risk factors. Secondary endpoints included the association between individual risk factors and LNM, as well as locoregional recurrences during active surveillance.

**Results:** A total of 103 patients (mean age 66 (SD 10), 56% male) with a median lesion size of 25 mm (IQR 15) were included. Endoscopic LE was performed in 31 patients (30%) and surgical LE in 72 (70%). After LE, 76 patients (74%) underwent cTME while 27 (26%) opted for active surveillance. The overall LNM rate was 17.5% (18/103). Among the 36 patients (35%) without histological risk factors, the LNM rate was 5.6% (2/36; OR 0.19, 95% CI 0.04‐0.87, *p* = 0.032). Both LNM cases were detected in the cTME group. LNM rates per individual risk factor are presented in Table 1.

In the active surveillance group, no locoregional recurrences occurred in the 10 patients without risk factors during a median follow‐up of 53 months (IQR 26). Among patients with risk factors, two of 14 patients with one risk factor developed a locoregional recurrence and one of two patients with two risk factors. All recurrences occurred intramurally following prior surgical LE. The only patient with three risk factors had no locoregional recurrence but died from unrelated cause at 24 months.

**TABLE 1**Univariate analyses of histological risk factors for lymph node metastasis.

**Table:** 

Risk factor	*n*/*N* (%)	Lymph node metastasis rate (%)	Odds ratio (95% CI)	*p*‐value
No histological risk factors	36/103 (35.0)	2/36 (5.6)	0.19 (0.04–0.87)	0.032
Lymphovascular invasion	47/103 (45.6)	12/47 (25.5)	7.0 (2.2–21.8)	0.055
Small vessel invasion	32/103 (31.1)	11/32 (34.4)	4.3 (1.5–12.5)	0.004
Large vessel invasion	28/103 (27.2)	4/28 (14.3)	7.5 (2.4–23.3)	0.604
Tumor budding and/or poorly differentiated clusters grade 2–3	36/103 (35.0)	13/36 (36.1)	7.0 (2.2–21.8)	< 0.001
Tumor budding grade 2–3	29/103 (28.2)	10/29 (34.5)	4.3 (1.5–12.5)	0.007
Poorly differentiated clusters grade 2–3	19/103 (18.4)	9/19 (47.4)	7.5 (2.4–23.3)	< 0.001
Adverse histology	13/103 (12.6)	6/13 (46.2)	5.6 (1.6–19.4)	0.007
Poor tumor differentiation	7/103 (6.8)	3/7 (42.9)	4.1 (0.8–20.0)	0.086
Mucinous subtype	6/103 (5.8)	3/6 (50.0)	5.5 (1.0–29.7)	0.049
Perineural invasion	11/103 (10.7)	5/11 (45.5)	5.1 (1.3–19.0)	0.016

**Conclusion:** In this group of pT2 rectal cancer patients treated with LE, detailed histological analysis suggests a low LNM risk (5.6%) in patients without histological risk factors. No locoregional recurrences occurred in this low‐risk group during active surveillance. As 35% of pT2 rectal cancers lacked histological risk factors, a risk‐stratified approach could potentially allow a substantial proportion of patients to avoid cTME. Prospective validation in larger cohorts is needed before clinical implementation.

**Disclosure:** Nothing to disclose.

## LB15 | Hologenomic Analysis of Rectal Mucus Sampling for Colorectal Cancer (Crc) and Advanced Adenomas: Oricol‐Egi‐02 Study Results (Nct04659590)

I. Daniels ^1,2^, E. Morales‐Walker^1^, K. Patel^1^, A. Tock^1^, C. Orthodoxou^1^, A. MacRitchie^1^, S. Njoroge^1^, E. Olaniru^1^, I. Mendes^3^, D. Baker^4^, A. Page^1^, H. Humphrey^2^, E. Walker^2^, F. McDermott^2^, S. Spencer^1^, S. Bird^5^, K.‐V. Savva^6^, C. Cunningham^5^, H. Rottenburg^7^, H. Sisodia^7^, N. Battersby^7^, G. Jones^8^, J. Lacy‐Colson^8^, A. Baggaley^6^, C. Peters^6^, A. Dodd^1^, K. Kang^1^, C. Hamon^1^, A. Crespillo‐Casado^1^, M. Sands^1^, H. Lywood^1^, D. Wise^1^

^1^Origin Sciences Ltd, Cambridge, UK, ^2^Department of Surgery, Royal Devon University Healthcare Trust, Exeter, UK, ^3^Theiagen Genomics, Denver, Colorado, USA, ^4^Quadram Institute, Norwich, UK, ^5^Department of Surgery, Oxford University Hospitals NHS Trust, Oxford, UK, ^6^Department of Surgery & Cancer, Imperial College, London, UK, ^7^Department of Surgery, Royal Cornwall NHS Trust, Truro, UK, ^8^Department of Surgery, Royal Shrewsbury Hospital, Shrewsbury, UK

**Contact E‐Mail Address:** ian.daniels@originsciences.com

**Introduction:** Colorectal cancer (CRC) is the third commonest malignancy globally and the second leading cause of cancer mortality. Prognosis is highly stage‐dependent, with survival exceeding 85% for early‐stage diagnoses but dropping sharply at advanced stages. While national screening programs have reduced mortality in older populations, incidence in younger individuals is rising, often accompanied by delays in diagnosis. Existing non‐invasive approaches, such as quantitative faecal immunochemical (qFiT) testing and blood‐based liquid biopsies, provide limited sensitivity, particularly for pre‐malignant or early‐stage disease, leaving colonoscopy as the invasive gold standard. Because CRC is a mucosal disease, rectal mucus represents an under‐explored biospecimen enriched for exfoliated cells, DNA, and microbial material, potentially enabling early and minimally invasive detection.

**Aims & Methods:** We assessed rectal mucus sampling as a novel diagnostic approach for CRC and adenomatous polyps. Using the OriCol device, rectal mucus was retrieved from 800 participants from 4 NHS colorectal surgical outpatients clinics without the need for bowel preparation (July 2020–October 2023). A hologenomic strategy was applied, integrating three complementary laboratory pipelines: error‐corrected next‐generation sequencing for somatic mutations (*n* = 161), enzymatic methyl‐sequencing for DNA methylation profiling (*n* = 208), and whole‐genome shotgun metagenomics for microbiome characterization (*n* = 379). Data underwent rigorous quality control, with feature selection and integrative multi‐omics analysis used to identify clinically informative biomarkers, stratify pathology by site and stage, and evaluate diagnostic potential.

**Results:** Distribution of CRC was 24% right‐colon, 23% hepatic‐splenic flexure, 18% left‐colon, 35% rectal. Somatic mutation analysis revealed pathogenic variants in well‐known CRC drivers including TP53, APC, KRAS, BRAF, ERBB2, FBXW7, PIK3CA and SMAD4, with mutation allele frequencies representing disease state. DNA methylation profiling demonstrated widespread hypermethylation at CpG islands near promoters and transcription start sites, particularly in SDC2, LRRC4, PPP2R5C and RNF217, with gradients of signal strength from rectal to right‐sided cancers and from CRC to advanced adenomas (> 10 mm ± HG dysplasia), to early polyps/normal controls. Microbiome profiling identified 94 species associated with CRC, including previously reported taxa such as *Fusobacterium nucleatum* and *Hungatella hathewayi*, alongside novel associations with *Clostridium scindens* and *Anaerotruncus colihominis*. Multi‐omics integration markedly improved diagnostic resolution, clustering CRC and advanced adenomas polyps separately from controls, with early‐stage polyps occupying an intermediate position.

**Conclusion:** We established rectal mucus as a rich biospecimen for minimally invasive CRC diagnostics. By combining host genetic and epigenetic markers with microbial signatures, the hologenomic approach provides sensitive detection of pre‐cancerous lesions and stratification by site/stage, outperforming single‐modality assays. Importantly, local mucosal sampling enabled discovery of novel microbial‐pathology associations not detectable in stool‐based studies. These findings demonstrate the translational potential of rectal mucus hologenomics as a scalable diagnostic platform, offering a non‐invasive alternative for triaging symptomatic patients and potentially extending to screening programmes. Further clinical validation is underway to refine performance metrics and benchmark against existing diagnostic standards (ORICOL‐EGI‐04 Study NCT06649123).

**References:** Complete references for this work are listed in the accepted Nature Communications paper.

**Disclosure:** 1. This work has been accepted for publication in Nature Communications and is currently embargoed whilst final editing is completed. A letter of confirmation of acceptance for publication can be provided.

2. The study is funded and performed through Origin Sciences Ltd with many of the staff holding share options in the company. The clinical research teams were provided with educational grants from Origin Sciences Ltd to fund the research and support research fellows and nurses.

3. Rectal mucus samples and tumor tissues were collected following UK Health Regulatory Authority (HRA) and Ethics Committee approvals (IRAS 263745, 19/EM/0266). Written informed consent was obtained from all donors. All samples were collected from patients enrolled in the ORI‐EGI‐02 study; Exploratory Study of Rectal Mucus for Diagnosing Disease (NCT04659590) from four NHS Hospitals.

4. The presenting author is employed by the NHS as a Consultant Colorectal Surgeon and is Chief Medical Officer, Origin Sciences and holds share options with the company.

## Latest news: From top to bottom 14:30‐15:30/Room A1

## LB16 | Impact of Combined Proton‐Pump Inhibitor and Rifaximin Therapy in Patients With Functional Dyspepsia: A Randomized Controlled Trial

L. Zhu, H. Guo, J. Gao, S. Lu, Z. Liu, C. Chen, K. Wang, L. Duan

Department of Gastroenterology, Peking University Third Hospital, Beijing, China

**Contact E‐Mail Address:** gaojq_pku@163.com

**Introduction:** Functional dyspepsia (FD) is associated with duodenal eosinophilia and gut microbiota dysbiosis. Proton pump inhibitors (PPIs), as a first‐line therapy, can suppress duodenal eosinophilic inflammation to ameliorate symptoms, yet the symptom relief rate is low. Rifaximin (RIF), an antibiotic known to modulate the gut microbiota, may offer an alternative therapeutic avenue. It is hypothesized that combining RIF with PPIs could more effectively alleviate FD symptoms and duodenal inflammation by targeting the gut microbiota.

**Aims & Methods:** A single‐center, open‐label, randomized controlled clinical trial was conducted on subjects who met the inclusion and exclusion criteria (Trial registration: ChiCTR2300069313). Ninety‐six patients were randomly assigned in a 1:1 ratio to either the PPI + RIF group or the PPI group. The follow‐up period extended to 4 weeks post‐treatment cessation. The primary endpoint was the symptom relief rate, while secondary endpoints included symptom response rate, duodenal eosinophil counts, and changes in the microbiota. The study hypothesis was that the clinical efficacy of PPI + RIF would be superior to that of PPI monotherapy.

**TABLE 1**

**Table:** 

	PPI + RIF	PPI	*Z* value	*p* value	PPI + RIF	PPI	*Z* value	*p* value
Symptom relief rate	ITT				PP			
Week 2	54.2% (26/48)	54.2% (26/48)	0.000	0.500	53.7% (22/41)	53.3% (24/45)	0.030	0.488
Week 4	72.9% (35/48)	58.3% (28/48)	1.522	0.064	76.7% (33/43)	56.8% (25/44)	2.020	**0.022**
4 weeks post‐treatment	77.1% (37/48)	47.9% (23/48)	3.095	**0.001**	78.6% (33/42)	48.9% (22/45)	3.036	**0.001**

Symptom response rate	ITT				PP			
Week 2	70.8% (34/48)	72.9% (35/48)	−0.227	0.590	68.3% (28/41)	73.3% (33/45)	−0.514	0.696
Week 4	81.3% (39/48)	77.1% (37/48)	−0.503	0.307	83.7% (36/43)	75.0% (33/44)	1.012	0.156
4 weeks post‐treatment	85.4% (41/48)	68.8% (33/48)	1.982	**0.024**	85.7% (36/42)	71.1% (32/45)	1.688	**0.046**

**Abbreviations:** ITT: the intention‐to‐treat analysis; PP: the per‐protocol analysis; PPI: Proton Pump Inhibitor; PPI + RIF: Proton Pump Inhibitor plus Rifaximin.

**Results:** A total of 96 patients were enrolled in the randomized controlled study. There were no statistically significant differences in baseline demographic data and clinical characteristics between the two groups. According to the intention‐to‐treat (ITT) analysis, the PPI + RIF group showed significantly higher rates of symptom relief (*p* = 0.001) and symptom response (*p* = 0.024) at 4 weeks post‐treatment. These findings were supported by the per‐protocol (PP) analysis, which revealed a superior symptom relief rate for the combination group at both 4 weeks of treatment (*p* = 0.022) and 4 weeks post‐treatment (*p* = 0.001), as well as a superior symptom response rate at 4 weeks post‐treatment (*p* = 0.046). Combination therapy also led to a greater reduction in duodenal eosinophil infiltration (*p* = 0.036) and significantly decreased peak hydrogen sulfide (H_2_S) levels (*p* < 0.001), indicating a reduction in small intestinal bacterial load. Oral microbiota analysis revealed that *β*‐diversity of the oral flora increased following the PPI + RIF intervention, with an increased relative abundance of *Enterococcus* (*p* = 0.004). Conversely, the PPI intervention led to the enrichment of the potentially pathogenic bacterium Catonella (*p* = 0.006). Notably, treatment responders in the PPI + RIF group showed a specific enrichment of the Eubacterium_yurii_group (*p* = 0.037), an alteration associated with the amelioration of duodenal eosinophilic inflammation and the enhancement of pathways related to preventing bacterial invasion of the epithelium.

**Conclusion:** In patients with FD, combining a PPI with Rifaximin is superior to PPI monotherapy, improving both symptoms and duodenal eosinophil infiltration. This effect is linked to a reduction in H_2_S‐producing bacteria and a beneficial shift in the oral microbiota. The oral abundance of *Eubacterium_yurii_group* may serve as a biomarker for treatment response.

**Disclosure:** Nothing to disclose.

## LB17 | A Novel Multi‐Epitope Based Vaccine Against *Helicobacter Pylori*

B. Kalali ^1,2^, H. Moeini^1,2^, A. Mostafazadeh^3^, A. Yadegar^4^, V. Wedershoven^1^, C. Schulz^2^, P. Malfertheiner^2^

^1^Iguana Biotechnology, Munich, Germany, ^2^Department of Medicine 2, Ludwig Maximilian University, Munich, Germany, ^3^Department of Immunology and Microbiology, Babol University of Medical Sciences, Babol, Iran, ^4^Research Institute for Gastroenterology and Liver, Shahid Beheshti Medical University, Tehran, Iran

**Contact E‐Mail Address:** bk@iguana-biotec.com

**Introduction:** *Helicobacter pylori* infection affects approximately half of the global population and may lead to severe complications, including gastric cancer. Current antibiotic‐based therapies face the challenge of rising resistance rates. Therefore, there is an urgent medical need for an effective vaccine. Despite extensive research efforts, no *H. pylori* vaccine has yet made it to clinical application. Aim of our study is the development of a novel multi‐epitope‐based *H. pylori* vaccine.

**Aims & Methods:** Sixteen selected B‐ and T‐cell epitopes from six key virulence factors were fused to create the Multi‐Epitope‐Unit (MEU) antigen. Two vaccine formulations were evaluated: MEU combined with flagellin as adjuvant, and MEU incorporated into the Modified Vaccinia virus Ankara (MVA) genome for enhanced antigen delivery. The efficacy was assessed in murine models using *H. pylori* SS1 infection, with evaluation in both prophylactic and therapeutic settings. To explore the possible clinical relevance, crude antigens from 48 human clinical isolates of *H. pylori* obtained from international healthcare centers were tested for binding capacity with anti‐MEU antibodies generated from immunized mice.

**Results:** Both vaccine formulations elicited robust MEU‐specific antibody and CD4+ T‐cell responses. The vaccines generated a balanced Th1/Th2 immune response profile and significantly elevated CD4+NKT‐like cell levels. Complete bacterial clearance was achieved with protein‐based vaccination followed by MVA boost or two doses of MVA vaccine in both prevention and treatment models. Binding assays revealed that anti‐MEU antibodies demonstrated positive binding capacity to crude antigens from all 48 tested human clinical isolates (100% reactivity), confirming broad‐spectrum recognition across diverse *H. pylori* strains from different geographical origins.

**Conclusion:** This study represents groundbreaking research, presenting a novel promising *H. pylori* vaccine candidate. The comprehensive testing against 48 human clinical isolates from international healthcare units provides unprecedented evidence of functional immune responses. Our data demonstrate 100% binding reactivity of anti‐MEU antibodies to crude antigens from *ALL* tested clinical isolates, representing diverse geographical origins and strain variations. This finding demonstrates for the first time that a *H. pylori* vaccine can generate immune responses effective against a vast spectrum of human pathogenic strains, which has eluded all previous vaccine approach failures and poses high expectation for a successful clinical application. Our multi‐epitope vaccine represents a novel *H. pylori* vaccine development, being the first to demonstrate both complete bacterial clearance in preclinical models as well as a broad‐spectrum reactivity against human clinical isolates. These results provide strong scientific rationale for advancing to toxicology studies and clinical evaluation, offering the first genuine hope for both prevention and treatment of *H. pylori* infections in human populations worldwide.

**Disclosure:** BK, HM and VW are employed by Iguana Biotechnology. The remaining authors declare no conflicts of interest.

## LB18 | Management of Severe Erosive Gastroesophageal Reflux Disease (EGERD) With High‐Dose Proton Pump Inhibitors: Interim Results From a Real‐World Observational Study (Power 3.0)

M. Goenka^1^, K. Pebbili ^2^, B. Namala^2^

^1^Department of Gastroenterology, Apollo Gleneagles Hospitals, Kolkata, India, ^2^Dr Reddys Laboratories Ltd, Hyderabad, India

**Contact E‐Mail Address:** kranthikiranpebbili@drreddys.com

**Introduction:** Treatment response in patients with severe erosive gastroesophageal reflux disease (eGERD) is often inadequate using regular doses of proton pump inhibitors (PPIs), thus necessitating escalation of dosing. High‐dose PPIs improve compliance in such patients, leading to better outcomes. However, comparative data on effectiveness of high‐dose PPIs is limited.

**Aims & Methods:** This interim analysis assessed symptomatic improvement of regurgitation in an ongoing, prospective, real‐world, observational study to compare the effectiveness of rabeprazole 40 mg to pantoprazole 80 mg and omeprazole 40 mg/esomeprazole 80 mg. Adult patients with endoscopically confirmed severe eGERD were enrolled. Patients shifted from low‐dose PPIs or started on high‐dose PPIs were included; those with complications of GERD were excluded.

**Results:** A total of 260 patients (rabeprazole, 130; omeprazole/esomeprazole, 65; pantoprazole, 65) from 37 sites in India were analyzed. Although symptomatic improvement of diurnal and nocturnal regurgitation based on patient‐reported severity scores was observed among the three arms after 4 ± 1 weeks of treatment, the complete‐resolution rate of diurnal regurgitation was significantly higher with rabeprazole (17.97%) compared to omeprazole/esomeprazole (5.08%; *p* = 0.018) and higher than pantoprazole (8.20%; *p* = 0.077). Similarly, complete resolution of nocturnal regurgitation was significantly higher with rabeprazole (14.06%) compared to omeprazole/esomeprazole (1.59%; *p* = 0.007) and higher than pantoprazole (7.94%; *p* = 0.221). Among patients with associated work‐related stress (*n* = 132), one‐fifth in the rabeprazole arm experienced complete resolution of diurnal regurgitation compared to none in the omeprazole/esomeprazole (*p* = 0.013) or pantoprazole (*p* = 0.030) arms. Patients with lack of regular physical exercise (83.08%) exhibited higher rate of complete resolution of nocturnal regurgitation with rabeprazole (13.73%) compared to the other PPIs. Overall, elderly patients > 60 years of age (mean ± SD, 68.82 ± 6.54 years) treated with rabeprazole showed significantly higher complete‐resolution rates of diurnal regurgitation compared to pantoprazole (36.67% vs. 0%; *p* = 0.003). For complete resolution of nocturnal regurgitation, rabeprazole proved to be significantly better when compared to omeprazole/esomeprazole (31.25% vs. 0%; *p* = 0.015) in elderly patients Table 1.

**Conclusion:** The interim results showed that rabeprazole led to significantly higher complete‐resolution rates of diurnal and nocturnal regurgitation at 4 ± 1 weeks of treatment than omeprazole/esomeprazole in adults with severe eGERD. In elderly patients, rabeprazole showed significant resolution over pantoprazole.

**Disclosure: *Study*** ***funded* *by:*** Dr. Reddy's Laboratories Ltd., Hyderabad, Telangana, India.

## LB19 | Cost‐Effectiveness of Risk‐Stratified Colorectal Cancer Screening Based on Polygenic Risk for Different Ethnic/Racial Populations

L. Mwangi ^1^, C. Bruck^1^, L. Hsu^2^, M. Thomas^2^, U. Peters^2^, I. Lansdorp‐Vogelaar^1^

^1^Erasmus MC, Public Health, Rotterdam, the Netherlands, ^2^Public Health Sciences Division, Fred Hutch Cancer Center, Seattle, Washington, USA

**Contact E‐Mail Address:** l.mwangi@erasmusmc.nl

**Introduction:** Colorectal cancer (CRC) remains a leading cause of cancer‐related deaths in the U.S., with persistent disparities across racial and ethnic groups. Current uniform screening guidelines overlook population differences in risk. Polygenic risk scores (PRS) offer a pathway to personalized, risk‐stratified screening, but their cost‐effectiveness across diverse populations is unclear.

**Aims & Methods:** In this study, we estimated the cost‐effectiveness of PRS‐based, risk‐stratified screening across four major U.S. racial and ethnic groups (White, Black, Asian and Hispanic), modeling lifetime costs, quality‐adjusted life‐years gained (QALYGs), and colonoscopy use to assess both overall and subgroup‐specific outcomes.

PRS data from the Genetics and Epidemiology of Colorectal Cancer Consortium (GECCO) were used to stratify individuals into risk 40 groups. The microsimulation model MISCAN‐Colon was used to determine the optimal colonoscopy screening strategy for each risk group within the racial and ethnic groups. Investigated strategies varied in terms of starting age (40–60 years), stopping age (70–85 years), and screening interval (5–15 years). Within each racial and ethnic group, the cost‐effectiveness of the optimal strategies of the 40 risk groups was compared with uniform colonoscopy screening (ages 45–75, every 10 years).

**Results:** Risk‐stratified screening produced different recommendations across risk levels and populations. For low‐risk groups, strategies ranged from no screening in Whites and Blacks to a one‐time colonoscopy at age 55 for Asians and at age 60 for Hispanics. In contrast, for high‐risk groups, all populations benefited most from intensive screening with colonoscopy every 5 years from ages 40–85. Across all racial and ethnic groups, PRS‐guided screening outperformed uniform strategies, preventing more CRC cases and deaths but at the cost of higher colonoscopy use and costs. In constrained analyses, when QALYGs were fixed to the level of uniform screening, risk‐stratified screening reduced costs by 6%–27% and when costs were fixed to the level of uniform screening QALYGs increased by 8%–29% These efficiency gains were most pronounced in Hispanics (−27% costs; +29% QALYGs) and Asians (−14% costs; +13% QALYGs), with smaller but consistent improvements in Whites (−9% costs; +12% QALYGs) and Blacks (−6% costs; +8% QALYGs).

**TABLE 1**Lifetime effects and costs per 1000 40‐year‐old individuals for no screening, uniform screening, and PRS‐guided risk‐stratified screening at a $100,000/QALYG threshold, with QALYGs‐ and cost‐constrained analyses, stratified by racial and ethnic group (results only for Hispanics and Asians) Asians

**Table:** 

Strategy	QALYGs	Costs	LYGs	Colonoscopies	CRC cases	CRC deaths
Uniform	2936	113,088	2297	69,207	471	128
Risk‐stratified WTP $100K	3841	137,490	2962	136,528	289	60
Constraining QALYGs WTP $30.8K	2934	99,201	2263	69,892	354	89
Constraining costs WTP $40.3K	3329	112,031	2568	99,562	291	65
Hispanics						
Uniform	2347	104,423	1854	64,953	402	109
Risk‐stratified WTP $100K	3073	126,058	2408	123,137	253	52
Constraining QALYGs WTP $25.4K	2342	77,087	1834	64,845	309	78
Constraining costs WTP $80.1K	3013	93,895	2359	117,402	256	54

**Conclusion:** PRS‐guided screening may offer a pathway to more tailored CRC prevention, with modest overall gains but potentially greater cost savings and health benefits in Hispanics and Asians.

**References**

1. J. M. Carethers, “Racial and Ethnic Disparities in Colorectal Cancer Incidence and Mortality,” *Advances in Cancer Research* 151 (2021): 197–229, 10.1016/bs.acr.2021.02.007.
2. F. P. May, L. Yang, E. Corona, B. A. Glenn, and R. Bastani, “Disparities in Colorectal Cancer Screening in the United States Before and After Implementation of the Affordable Care Act,” *Clinical Gastroenterology and Hepatology* 18, no. 8 (2020): 1796–1804.e2, 10.1016/j.cgh.2019.09.008.
3. P. Dixon, E. Keeney, J. C. Taylor, S. Wordsworth, and R. M. Martin, “Can Polygenic Risk Scores Contribute to Cost‐Effective Cancer Screening? A Systematic Review,” *Genetics in Medicine* 24, no. 8 (2022): 1604–1617, 10.1016/j.gim.2022.04.020.
4. M. Tamlander, B. Jermy, T. T. Seppälä, et al., “Genome‐Wide Polygenic Risk Scores for Colorectal Cancer Have Implications for Risk‐Based Screening,” *British Journal of Cancer* 130, no. 4 (2024): 651–659, 10.1038/s41416‐023‐02536‐z.

**Disclosure:** No conflicts of interest.

## LB20 | Natural Language Processing Based Classification of Bowel Preparation Quality and Its Association With Adenoma Detection in > 11,000 Colonoscopy Reports: A Late‐Breaking Real‐World Analysis

B. F. Ağargün, S. Genç Uluçeçen, G. Dağcı, M. A. Yagli, A. Gurbanov, P. Telli, S. Mammadov, E. Bilgin, C. Kiziltas, G. Kurular, A. Akın, B. Bozan, A. Cifcibasi Ormeci, F. Akyüz, K. Demir, F. Besisik, S. Kaymakoglu, B. Cavus

Department of Internal Medicine, Istanbul Faculty of Medicine, Istanbul University, Istanbul, Türkiye

**Contact E‐Mail Address:** bfagargun@istanbul.edu.tr

**Introduction:** Bowel preparation quality is a key determinant of colonoscopy effectiveness and adenoma detection [1, 2]. Manual evaluation of free‐text colonoscopy reports is labor‐intensive and limits quality monitoring [3]. Developing a natural language processing (NLP) application for measuring colonoscopy quality demonstrates the potential to automate this process by extracting key quality indicators [4].

**Aims & Methods:** We aimed to develop and validate a NLP approach to automatically classify bowel cleanliness and to comprehensively structure colonoscopy reports by extracting procedural and pathological variables. A total of 11,374 reports from our institutional endoscopy unit were analyzed. An annotated dataset of 1018 reports labeled as “good,” “intermediate,” or “poor” preparation was used to train a TF‐IDF–based logistic regression classifier. Cross‐validation and test performance were evaluated using accuracy, precision, recall, and F1‐scores, and the model was applied to the full dataset. Additional variables (cecal intubation, polyp presence, number, size, localization, polypectomy method, and hemostasis application) were extracted using rule‐based NLP heuristics. Adenoma detection rate (ADR) was calculated in a predefined subset (January 2023–July 2025) with complete pathology data, and multivariable logistic regression identified independent predictors.

**Results:** The NLP classifier achieved a cross‐validated accuracy of 92%. Precision and recall were highest for “good” preparation (0.98 and 0.99) and acceptable for “intermediate” (0.85 and 0.82) and “poor” (0.85 and 0.87). Application to the full cohort yielded 5787 (50.9%) good, 2176 (19.1%) intermediate, and 3411 (30.0%) poor preparations. Leveraging these structured data, ADR analysis in the subset of 5420 patients showed an overall rate of 20.4%. Multivariable analysis demonstrated that ADR was independently associated with older age (OR 1.05 per year, 95% CI 1.05–1.06, *p* < 0.001), male sex (OR 1.74, 95% CI 1.51–2.01, *p* < 0.001), adequate bowel preparation (OR 1.46, 95% CI 1.21–1.75, *p* < 0.001), cecal intubation (OR 1.76, 95% CI 1.43–2.16, *p* < 0.001), and procedures performed by specialists compared with trainees (24.0% vs. 20.3%; OR 1.25, 95% CI 1.05–1.47, *p* = 0.010).

**TABLE 1**

**Table:** 

Variable	Value/*N*	Precision/recall	ADR%	Multivariable OR (95% CI)	*p*‐value
Cohort distribution (*n* = 11,374)	Good: 5787 (50.9%)	0.98/0.99	—	—	—
	Intermediate: 2176 (19.1%)	0.85/0.82	—	—	—
	Poor: 3411 (30.0%)	0.85/0.87	—	—	—
ADR analysis subset (*n* = 5420)					
Age (per year)	—	—	—	1.05 (1.05–1.06)	< 0.001
Male sex (ref: female)	2661 versus 2759	—	23.8 versus 17.1	1.74 (1.51–2.01)	< 0.001
Bowel preparation (good/intermediate vs. poor)	3891 versus 1529	—	22.1 versus 16.0	1.46 (1.21–1.75)	< 0.001
Cecal intubation (yes vs. no)	4209 versus 1211	—	21.0 versus 16.5*	1.76 (1.43–2.16)	< 0.001
Operator (specialist vs. trainee)	1045 versus 4149	—	24.0 versus 20.3	1.25 (1.05–1.47)	0.010

*ADR% for cecal intubation groups calculated from subset.

**Conclusion:** This large‐scale real‐world analysis demonstrates the feasibility of NLP‐based automated classification of bowel preparation quality and its direct utility in generating structured colonoscopy datasets for quality monitoring. By integrating bowel cleanliness with other performance indicators (age, sex, cecal intubation, operator expertise), our framework not only achieved high classification accuracy but also provided clinically relevant benchmarks for ADR, supporting targeted quality improvement in endoscopy units.

**References**

1. B. T. Clark, P. Protiva, A. Nagar, et al., “Quantification of Adequate Bowel Preparation for Screening or Surveillance Colonoscopy in Men,” *Gastroenterology* 150, no. 2 (2016): 396–405, 10.1053/j.gastro.2015.09.041.
2. B. T. Clark, T. Rustagi, and L. Laine, “What Level of Bowel Prep Quality Requires Early Repeat Colonoscopy: Systematic Review and Meta‐Analysis of the Impact of Preparation Quality on Adenoma Detection Rate,” *American Journal of Gastroenterology* 109, no. 11 (2014): 1714–1724, 10.1038/ajg.2014.232.
3. G. S. Raju, P. J. Lum, R. S. Slack, et al., “Natural Language Processing as an Alternative to Manual Reporting of Colonoscopy Quality Metrics,” *Gastrointestinal Endoscopy* 82, no. 3 (2015): 512–519, 10.1016/j.gie.2015.01.049.
4. H. Harkema, W. W. Chapman, M. Saul, E. S. Dellon, R. E. Schoen, and A. Mehrotra, “Developing a Natural Language Processing Application for Measuring the Quality of Colonoscopy Procedures,” supplement, *Journal of the American Medical Informatics Association* 18, no. S1 (2011): S150–S156, 10.1136/amiajnl‐2011‐000431.

**Disclosure:** Nothing to disclose.

